# Impaired CK1 Delta Activity Attenuates SV40-Induced Cellular Transformation *In Vitro* and Mouse Mammary Carcinogenesis *In Vivo*

**DOI:** 10.1371/journal.pone.0029709

**Published:** 2012-01-03

**Authors:** Heidrun Hirner, Cagatay Günes, Joachim Bischof, Sonja Wolff, Arnhild Grothey, Marion Kühl, Franz Oswald, Florian Wegwitz, Michael R. Bösl, Anna Trauzold, Doris Henne-Bruns, Christian Peifer, Frank Leithäuser, Wolfgang Deppert, Uwe Knippschild

**Affiliations:** 1 Department of General-, Visceral- and Transplantation Surgery, University of Ulm, Ulm, Germany; 2 Institute of Molecular Medicine and Max-Planck-Research Group on Stem Cell Aging, University of Ulm, Ulm, Germany; 3 Department of Tumor Virology, Heinrich-Pette-Institute, Leibniz-Center for Experimental Virology, Hamburg, Germany; 4 Department of Internal Medicine I, University of Ulm, Ulm, Germany; 5 Max Planck Institute of Neurobiology Transgenic Mouse Models, Max Planck Institute, Martinsried, Germany; 6 Division of Molecular Oncology, Institute for Experimental Cancer Research, CCCNorth, UK S-H, Kiel, Germany; 7 Institute for Pharmacy, University of Kiel, Kiel, Germany; 8 Department of Pathology, University of Ulm, Ulm, Germany; Clermont Université, France

## Abstract

Simian virus 40 (SV40) is a powerful tool to study cellular transformation *in vitro*, as well as tumor development and progression *in vivo*. Various cellular kinases, among them members of the CK1 family, play an important role in modulating the transforming activity of SV40, including the transforming activity of T-Ag, the major transforming protein of SV40, itself. Here we characterized the effects of mutant CK1δ variants with impaired kinase activity on SV40-induced cell transformation *in vitro*, and on SV40-induced mammary carcinogenesis *in vivo* in a transgenic/bi-transgenic mouse model. CK1δ mutants exhibited a reduced kinase activity compared to wtCK1δ in *in vitro* kinase assays. Molecular modeling studies suggested that mutation N172D, located within the substrate binding region, is mainly responsible for impaired mutCK1δ activity. When stably over-expressed in maximal transformed SV-52 cells, CK1δ mutants induced reversion to a minimal transformed phenotype by dominant-negative interference with endogenous wtCK1δ. To characterize the effects of CK1δ on SV40-induced mammary carcinogenesis, we generated transgenic mice expressing mutant CK1δ under the control of the *whey acidic protein* (WAP) gene promoter, and crossed them with SV40 transgenic WAP-T-antigen (WAP-T) mice. Both WAP-T mice as well as WAP-mutCK1δ/WAP-T bi-transgenic mice developed breast cancer. However, tumor incidence was lower and life span was significantly longer in WAP-mutCK1δ/WAP-T bi-transgenic animals. The reduced CK1δ activity did not affect early lesion formation during tumorigenesis, suggesting that impaired CK1δ activity reduces the probability for outgrowth of *in situ* carcinomas to invasive carcinomas. The different tumorigenic potential of SV40 in WAP-T and WAP-mutCK1δ/WAP-T tumors was also reflected by a significantly different expression of various genes known to be involved in tumor progression, specifically of those involved in *wnt*-signaling and DNA repair. Our data show that inactivating mutations in CK1δ impair SV40-induced cellular transformation *in vitro* and mouse mammary carcinogenesis *in vivo*.

## Introduction

Viral and cellular oncogenes both induce a stepwise deregulation of the cellular gene expression program, leading to perturbation of the cell cycle and of normal cell growth and, in the end, to cellular transformation and tumor development. However, it is becoming increasingly clear that, in addition, cellular co-factors play an important role in this process, among them protein kinases, as they can modulate the oncogenic activity of proteins involved in tumorigenesis [1], [2]. As an example, members of the casein kinase 1 (CK1) family have been shown to modulate the activity of various tumor suppressors and oncoproteins [3]–[10]. In this regard, transformation-relevant phosphorylation sites of simian virus 40 (SV40) large tumor antigen (T-Ag) have been identified which are targeted by CK1 isoforms *in vitro* [11]–[14].

CK1δ, a member of the CK1 kinase family and the mammalian counterpart of yeast Hrr25, is involved in the regulation of many different cellular processes, including cell proliferation and cell death [8], [15]. Mutations and alterations in the expression and/or activity of CK1δ have been detected in various tumor entities, e.g. in adenocarcinomas of the pancreas [16], in mammary tumors [15] and in adenoid cystic carcinomas [17], suggesting that changes in CK1δ activity can contribute to carcinogenesis. As SV40 mediated transformation is a well established model to study cellular factors associated with the transformation process, we characterized the role of CK1δ in SV40 mediated transformation in a cell culture system and in the WAP-T transgenic mouse model [18]–[21].

SV40 wild-type transformed cells (SV-52) and cellular revertants (Rev2) derived from them are well characterized in regard to T-Ag and p53 expression and functions [11], [22]. Therefore, this matched pair of cells is a valuable tool to analyze cellular factors that influence the transforming activity of T-Ag *in vitro*. SV-52 cells display a so called maximal transformed phenotype and were established after microinjection of SV40 DNA into rat REF52 cells; Rev2 cells are T-Ag positive flat revertants of SV-52 cells expressing wild-type T-Ag with regard to its sequence [23]. However, T-Ag expressed in Rev2 cells shows an impaired transforming activity that correlates with a reduced ability to bind to SV40 *ori*-DNA *in vitro* and to associate with the cellular chromatin *in vivo*. Furthermore, Rev2 T-Ag revealed a reduced phosphorylation at specific transformation-relevant serine and threonine residues [11], [24]. Also phosphorylation of the p53 protein associated with Rev2 T-Ag is altered [25], suggesting that the altered phosphorylation state of the T-Ag/p53 complex is causally involved in causing the revertant phenotype of Rev2 cells [11].

WAP-T mice, a model for oncogene induced mammary carcinogenesis [18]–[21], allow to investigate the role of cellular factors influencing the activity of T-Ag in SV40-induced tumorigenesis *in vivo*. In adult female WAP-T mice activation of the transgene, the SV40 early gene region flanked by a ∼1.4 kb upstream region of the gene coding for the mouse *whey acidic protein* (WAP) [18], [21], is initiated during late pregnancy in mammary epithelial cells concomitantly with the endogenous WAP gene [26], [27]. Expression of alternatively spliced SV40 early mRNAs coding for T-Ag, small t-antigen (st), and the 17 kT protein [28] drives mammary carcinogenesis by mimicking a variety of genetic alterations commonly seen in human breast carcinomas, like abrogation of the pRb-controlled G1-checkpoint, and inactivation of the tumor suppressor p53 [29]. As a consequence of SV40 early gene expression, WAP-T mice develop multiple alveolar lesions - multifocal intraepithelial neoplasia (MIN) - after mammary gland involution. Some of these focal lesions further progress to invasive, but rarely metastatic mammary adenocarcinomas [20], ranging from a well to a poorly differentiated phenotype [19]. The relevance of this model is emphasized by the close similarity in histology of the mouse tumors with corresponding human tumors [30] and by cross-species match of WAP-T tumors with human basal-like breast tumors (manuscript submitted).

We here demonstrate that a reduced CK1δ activity impairs SV40-induced cell transformation *in vitro* and mammary tumorigenesis *in vivo*. Introduction of CK1δ mutants with a reduced kinase activity into SV40 maximal transformed cells (SV-52 cells) reversed their phenotype to minimal transformants. Although invasive tumor formation was observed upon transgene induction in both, WAP-T and WAP-mutCK1δ/WAP-T mice, WAP-mutCK1δ/WAP-T bi-transgenic mice showed a prolonged survival compared to WAP-T mice. Furthermore, we found that the expression of genes coding for proteins involved in *wnt*-signaling and DNA repair was significantly different between tumors of WAP-T and WAP-mutCK1δ/WAP-T mice. These genes are known to be involved in tumor progression and their expression is influenced by products of the SV40 early region and by CK1δ, respectively [31]–[38]. We conclude that the reduced activity of mutant CK1δ variants attenuates SV40 mediated cellular transformation *in vitro* and SV40-induced mouse mammary carcinogenesis *in vivo*.

## Materials and Methods

### Cell culture

REF52 fibroblasts [23], SV-52 cells (a SV40 transformed cell line established after microinjection of SV40 DNA into REF52 cells; [23]), Rev2 cells (a T-Ag positive, flat revertant of SV-52 cells; [22]), SV-CK1δ(rev) and SV-mutCK1δ cells were maintained in Dulbecco's modified Eagle's medium (DMEM) containing 10% heat-inactivated fetal calf serum (FCS) (Gibco BRL, Karlsruhe, Germany) in a humidified 5% CO_2_ atmosphere.

### Cloning in soft agar

1×10^3^, 5×10^3^ and 1×10^4^ cells per 35 mm-diameter dish were plated in duplicates in DMEM containing 10% FCS and 0.3% agar (Bacto Agar; Difco Laboratories, Heidelberg, Germany) onto a bottom layer of 0.5% agar in DMEM. Colonies were scored and photographed 20 days after plating.

### Fluorescence microscopy

Cells were grown on coverslips for 2 days at 37°C, fixed in 3% formaldehyde in PBS, containing 1 mM CaCl_2_ and 0.05 mM MgCl_2_ for 10 min at 37°C, permeabilized in PBS containing 0.3% Triton X-100 at 37°C for 3 min and treated with PBS containing 0.2% gelatine for 45 min. Staining was done according to Wulf et al. [39] with TRITC-phalloidin (0.05 mg/ml; Sigma-Aldrich, Munich, Germany) and afterwards, cells were mounted on slides. Fluorescence microscopy was performed using an Olympus IX81 microscope in combination with the Cell^R^ Imaging Software (Olympus, Hamburg, Germany).

### Cell lysis

Cells were washed in ice-cold PBS and lysed either in sucrose lysis buffer (20 mM Tris-HCl [pH 7.0], 0.27 M sucrose, 1 mM EDTA, 1 mM EGTA, 1% Triton X-100, 1 mM benzamidine, 4 µg/ml leupeptin, 30 µg/ml aprotinin, 0.1% β-mercaptoethanol) or in NP40 lysis buffer (1% NP-40, 50 mM Tris-HCl [pH 8.0], 150 mM NaCl, 10% glycerol, 5 mM DTT, 1 mM EDTA, 1 mM EGTA, 50 µM leupeptin and 30 µg/ml aprotinin).

### Fractionation of cellular extracts

SV-52, Rev2, SV-CK1δ(rev), SV-mutCKδ cells, as well as mammary tumor tissue of WAP-T and WAP-mutCK1δ/WAP-T mice were lysed in sucrose lysis buffer and fractionation was carried out as described elsewhere [40]. Briefly, cleared cell lysates were passed through 0.40 µm pore-size filters and 3 mg of total protein was applied to an anion exchange column (Resoure Q) attached to an Ettan LC purifier (GE Healthcare, Munich, Germany). The proteins were eluted with a linear ascending NaCl gradient.

### Overproduction and purification of recombinant proteins

The production and purification of the glutathione-S-transferase (GST) fusion proteins GST-p53^1–64^ (FP267), GST-wtCK1δ (FP449), GST-CK1δ(rev) (FP708), GST-mutCK1δ (FP1124) and baculovirus expressed T-Ag were carried out as described elsewhere [41], [42].

### *In vitro* kinase assays

*In vitro* kinase assays were carried out as described previously [40] using the GST-p53^1–64^ fusion protein FP267 or baculovirus expressed T-Ag as substrates, and a C-terminally truncated CK1δ (CK1δKD; NEB, Frankfurt a. M., Germany), or single fractions of fractionated cell or mammary tumor tissue extracts as sources of enzyme. The kinase activity in kinase peak fractions was also analyzed in the presence of CK1 specific inhibitors IC261 [43] or compound 17, which inhibits specifically CK1δ in the lower nanomolar range [44]. Phosphorylated proteins were separated by SDS-PAGE and the protein bands were visualized on dried Coomassie stained gels by autoradiography. Where indicated, the phosphorylated protein bands were excised and quantified by Cherenkov counting.

### Western blot analysis

To detect CK1δ in FPLC fractions, proteins were separated on SDS gels, transferred to Hybond-XL membranes (GE Healthcare, Munich, Germany) and probed with the CK1δ specific monoclonal antibody 128A (kindly provided by ICOS Corporation, Washington, USA). Detection was carried out using horseradish peroxidase-conjugated anti-mouse IgG as a secondary antibody, followed by chemiluminescence detection (ECL; GE Healthcare, Munich, Germany).

### Animals

All mice were housed and handled in accordance to official regulations for care and use of laboratory animals (UKCCCR Guidelines for the Welfare of Animals in Experimental Neoplasia). Ethical approval of all mouse experiments was granted by the Regierungspräsidium Tübingen (permission numbers 752, 904 and 1036). Transgenic mice were kept under barrier conditions with a 12 h light/dark cycle and access to food and water *ad libitum*. Male BALB/c WAP-mutCK1δ, mono-transgenic strain (mutCK1δ transgenic; backcross generation 11) and female BALB/c WAP-T mono-transgenic strain (T-Ag transgenic, line NP8, [20]) were interbred to obtain WAP-mutCK1δ/WAP-T bi-transgenic mice. In order to induce mutCK1δ expression in mammary glands, transgenic females were mated. The transgene expression was analyzed at different days of lactation (the date of birth was counted as day 1 of lactation). Age-matched non transgenic littermates served as controls. Mice were euthanized by CO_2_ and mammary glands, liver and spleen were eviscerated. Tissues were either snap-frozen and stored at −80°C or fixed in 4% formaldehyde containing 1% acetic acid. WAP-T transgenic and WAP-mutCK1δ/WAP-T bi-transgenic mice were sacrificed when they exhibited signs of morbidity, or when the tumor size exceeded 1.5 cm.

### RNA extraction and analysis

Total RNA was isolated by homogenization of frozen tissue with a homogenizer using the RNeasy Lipid Tissue Kit (Qiagen, Hilden, Germany). 1 µg of total RNA was used for reverse transcription using the RT^2^ First Strand Kit (SuperArray SABioscience, Karlsruhe, Germany) as described by the manufacturer. To check the quality of the cDNA a PCR was performed using the following primers to amplify the β-actin gene: 5′actin-R primer 5′-GTCAGGCAGCTCGTAGCTCT-3′ and 3′actin-L primer 5′-GGCATCCTCACCCTGAAGTA-3′. To exclude a possible genomic DNA contamination control PCRs were performed using the same cDNA used for screening the mice and the following primers: 5′actin-N primer 5′-CGAGCAGGAGATGGCCACTGC-3′ and 3′actin-H primer 5′-GTGAGCTCTCTGGGTGCTGGG-3′. The actin-H primer binds in the intron of the β-actin gene, whereas the actin-N primer binds in the exon of the gene.

### Gene expression analysis

Total RNA was isolated from frozen tissue using the peqGOLD RNAPure™ (Peqlab, Erlangen, Germany) protocol as described by the manufacturer. 1 µg isolated RNA was transcribed into cDNA using the RT^2^ First Strand Kit (SuperArray SABiosience, Karlsruhe, Germany). Gene profiling was done as described by the manufacturer using the RT^2^ profiler PCR arrays “mouse *wnt*-signaling pathway” and “mouse DNA repair” (each 84 genes). The reactions were performed on the Applied Biosystems 7500 Fast-Real Time PCR System (Applied Biosystems, Carlsbad USA). The results were read out with the 7500 Fast System SDS Software.

### Evaluation of the epithelial area fraction in mammary gland of WAP-T and WAP-mutCK1δ/WAP-T mice

Paraffin sections of WAP-T and WAP-mutCK1δ/WAP-T mammary glands obtained at day 60 post partum were stained with H&E and photographed with a 10× magnification. Pictures were then analyzed with the ImageJ software (v.1.43u; NIH, Bethesda, USA). Epithelial areas were selected using the “Polygon selection” tool and the respective surface measured by the function “Measure”. Using the same procedure, total mammary gland surface was measured for each picture. Finally, epithelial area fractions were estimated over the total mammary gland surface in Excel (Excel® 2007; Microsoft, Redmond, USA) and summarized in a graph with GraphPad Prism 5 (v5.03; GraphPad Software, La Jolla, USA). The histogram shows the mean of epithelial area fraction with standard error of the mean.

### Immunohistochemistry

Formalin fixed tissues were then dehydrated in a graded ethanol series, cleared in methyl benzoate, and embedded in paraffin. Sections were cut at 1 µm and mounted on glass slides. Staining procedures included deparaffinization in xylene, rehydration via transfer through graded alcohols and inhibition of endogenous peroxidase activity (Peroxidase Blocking Reagent; DAKO, Glostrup, Denmark). The sections were treated with the antigen retrieval solution Citra Plus, pH 6.03 (BioGenex, San Ramon, CA, USA) in a microwave oven, according to the manufacturer's instructions. For immunohistochemical detection of T-Ag or myc-mutCK1δ sections were incubated overnight at 4°C with the rabbit polyclonal T-Ag specific antiserum R15 (1∶5000; [20]) or with a c-myc specific antibody (A-14, 1∶600; Santa Cruz, Santa Cruz, USA), respectively. After washing in Tris-HCl buffer appropriate peroxidase conjugated secondary antibodies (N-Histofine®; Nichirei Corporation, Tokio, Japan) were applied at room temperature for 30 minutes. The enzymatic reaction was developed in a freshly prepared solution of 3,3′-diaminobenzidine using DAKO Liquid DAB Substrate-Chromogen solution. Finally, the sections were counterstained with hematoxylin and permanently mounted in Entellan (Merck, Darmstadt, Germany). Positive and negative controls were included for each case.

### Molecular Modeling

Modeling was performed on a DELL T5500 workstation (DELL, Round Rock, USA) using Schrödinger Suite Maestro 9.1 (Schrödinger, Portland, USA). A high quality homology model of CK1δ possessing rat sequence to match biochemical data of this study was generated based on PDB 1CSN [45] (origin from fission yeast, sequence identity 99%), containing Mg-ATP as ligand in the active site (model: wtCK1δ). Mutations CK1δ(rev): side chains of amino acids 24 (Tyr→Cys), 47 (Pro→Ser), 172 (Asn→Asp), 202 (Val→Ala); mutCK1δ: 24 (Tyr→Cys), 47 (Pro→Ser), 172 (Asn→Asp), 201 (Tyr→His), 202 (Val→Ala) 224 (Lys→Arg), 271 (Gln→Arg) were introduced. Subsequently the systems were minimized, respectively, using OPLS-2005 force field by default settings implemented in Schrödinger software package. Molecular surface of protein structures were calculated and represented as electrostatic surface.

### Statistical methods

For the analysis of the survival of WAP-T and WAP-mutCK1δ/WAP-T mice the total survival curves (Kaplan-Meier) were compared using the log-rank test. To demonstrate differences in the relative quantification of genes expressed in tumors of six different WAP-T and WAP-mutCK1δ/WAP-T mice the Mann-Whitney-U test was used. All statistical calculations were performed using the PASW Statistics 19.0 software (IBM, Ehningen, Germany).

### Additional methods

Information regarding the use of retroviral vectors, infection and transfection of cells, the cell fusion method, metabolic labelling of cells with [^35^S]-methionine, isolation of genomic DNA, Southern blot analysis, the *in situ* fractionation of cells, the construction of the WAP-CK1δ(rev) expression vector, the generation and screening of WAP-CK1δ(rev) transgenic mice, reverse transcription PCR (RT-PCR), the cloning of mutCK1δ, generation of CK1δ^N172D^ and the clinical tumor staging and histological tumor grading were provided in the supplementary data file S1 (additional methods).

## Results

### CK1δ phosphorylates SV40 T-Ag *in vitro*

**Figure 1:**
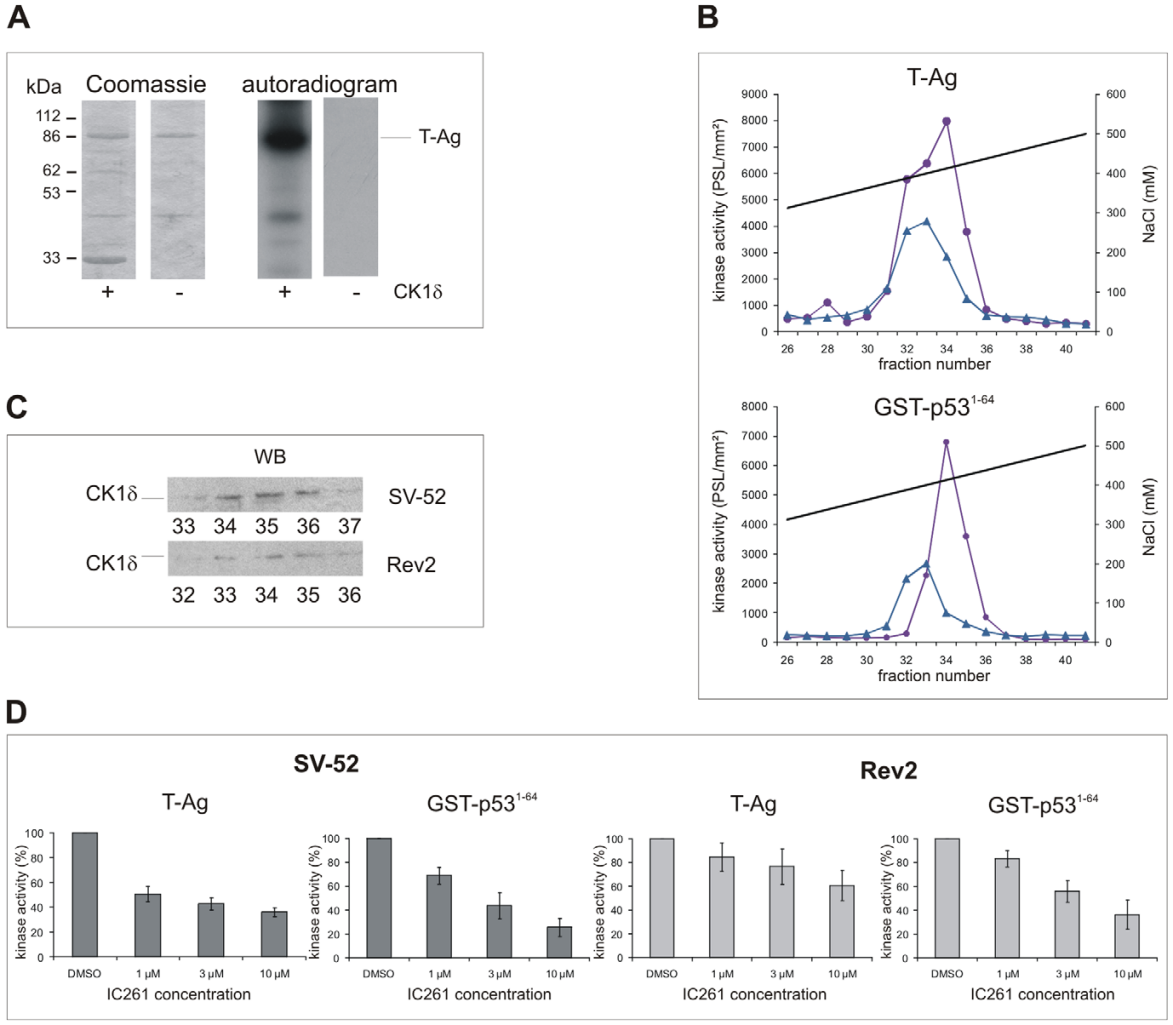
Characterization of the CK1 activity present in SV-52 and Rev2 cells. **(A) Phosphorylation of T-Ag by CK1δKD.** *In vitro* kinase assays were performed using baculovirus-expressed T-Ag as a substrate and a C-terminally truncated CK1δ (CK1δKD) as enzyme. The phosphorylated proteins were separated by SDS-PAGE (12.5%) and visualized by Coomassie staining. The degree of phosphorylation was documented by autoradiography. Addition of C-terminally truncated CK1δ is indicated by + or −. kDa: kilo dalton. **(B) Detection of CK1 activity in fractions derived from anion exchange chromatography.** Soluble extracts of SV-52 and Rev2 cells were prepared and equal amounts of protein were loaded onto a 1 ml Resource Q column. The proteins were eluted with a linear gradient of increasing NaCl concentration. 0.25 ml fractions were collected, and kinase activity was determined as described in Materials and Methods. The kinase activities in the peak fractions of SV-52 and Rev2 cells were determined using either T-Ag or GST-p53^1–64^ as a substrate. SV-52 cells: purple, closed circles; Rev2 cells: blue, closed triangles; — mM NaCl. **(C) Detection of CK1 in kinase peak fractions.** Western blot analyses were performed using proteins from the peak fractions of fractionated SV-52 and Rev2 cell lysates as described in Materials and Methods. CK1δ was detected using the CK1δ specific mouse monoclonal antibody 128A. **(D) Inhibition of CK1 kinase activity in SV-52 and Rev2 cellular extracts using the CK1 specific inhibitor IC261.** *In vitro* kinase assays were performed in the presence of 1 µM, 3 µM and 10 µM of IC261 using cellular fractions from fractionated SV-52 and Rev2 protein lysates as source of kinase. Phosphate incorporation into T-Ag and GST-p53^1–64^, respectively, was normalized towards DMSO control reactions.

The transforming activity of SV40 T-Ag is strongly influenced by site-specific phosphorylation [46]. Thus changes in the activity of cellular kinases targeting T-Ag could alter its transformation competence. Members of the casein kinase 1 (CK1) family are able to phosphorylate transformation-relevant phosphorylation sites of T-Ag *in vitro* [13], [14], [47], [48]. In minimal transformed Rev2 cells T-Ag complexed to p53 is underphosphorylated at transformation-relevant phosphorylation sites [11] which are targeted by CK1 isoforms *in vitro* [12]–[14], [48]. Since CK1δ phosphorylates p53 *in vivo* and co-immunoprecipitates with T-Ag/p53 complexes, it is most likely that CK1δ also targets T-Ag within the T-Ag/p53 complex [8], [49]–[56]. Indeed, *in vitro* kinase assays revealed that CK1δ is able to phosphorylate baculovirus-expressed T-Ag (figure 1A), suggesting that the altered phosphorylation of T-Ag and p53 [57] in Rev2 cells might result from an altered CK1δ activity. We therefore analyzed the activity of CK1δ in fractionated extracts of SV40 transformed SV-52 (maximal transformed) and Rev2 cells (minimal transformed). The kinase activity in fractionated Rev2 and SV-52 cell extracts, eluting at 421 mM and 434 mM NaCl, respectively (figure 1B), was reduced 2-fold in Rev2 cells compared to that in SV-52 cells when T-Ag was used as substrate, and approximately 3-fold when the GST-p53^1–64^ fusion protein was used as substrate (figure 1B). The detection of CK1δ in the kinase peak fractions by Western blot analyses (figure 1C) and inhibition of the kinase activity present in the kinase peak fractions by the CK1 specific small molecule inhibitor IC261 (figure 1D) confirmed that CK1δ is the main kinase present in the kinase peak fractions.

### A single mutation within the kinase domain of CK1δ(rev) and mutCK1δ is responsible for their reduced kinase activity

Sequencing of CK1δ cDNA isolated from Rev2 cells (CK1δ(rev)) revealed several point mutations, resulting in amino acid exchanges of amino acids 24 (Tyr→Cys), 47 (Pro→Ser), 172 (Asn→Asp), 202 (Val→Ala), 332 (Gly→Ser) and 384 (Ser→Pro) (table 1 and [58]). To elucidate the possible impact of these mutations on CK1δ activity, we performed molecular modeling studies. In these analyses, we included additional mutations at base pairs 601 (CAC→TAC), 671 (AAG→AGG) and 812 (CAG→CGG), leading to amino acid mutations at positions 201 (Try→His), 224 (Lys→Arg) and 271 (Gln→Arg) (table 1), which occurred in CK1δ(rev) after introduction of the CK1δ(rev) gene as a transgene into mice. These mutations were identified by sequencing of the transgene of the respective WAP-CK1δ(rev) mice (further referred to as mutCK1δ and WAP-mutCK1δ mice, respectively, see below).

**Table 1:** Point mutations and amino acid exchanges in CK1δ (rev) and mutCK1δ.

CK1δ(rev)		mutCK1δ	
position/point mutation	position/amino acid exchange	position/point mutation	position/amino acid exchange
71 (TAT→TGT)	24 (Tyr→Cys)	71 (TAT→TGT)	24 (Tyr→Cys)
139 (CCT→TCT)	47 (Pro→Ser)	139 (CCT→TCT)	47 (Pro→Ser)
514 (AAC→GAC)	172 (Asn→Asp)	514 (AAC→GAC)	172 (Asn→Asp)
605 (GTG→GCG)	202 (Val→Ala)	605 (GTG→GCG)	202 (Val→Ala)
994 (GGC→AGC)	332 (Gly→Ser)	994 (GGC→AGC)	332 (Gly→Ser)
1150 (TCT→CCT)	384 (Ser→Pro)	1150 (TCT→CCT)	384 (Ser→Pro)
		601 (CAC→TAC)	201 (Tyr→His)
		671 (AAG→AGG)	224 (Lys→Arg)
		812 (CAG→CGG)	271 (Gln→Arg)

**Figure 2:**
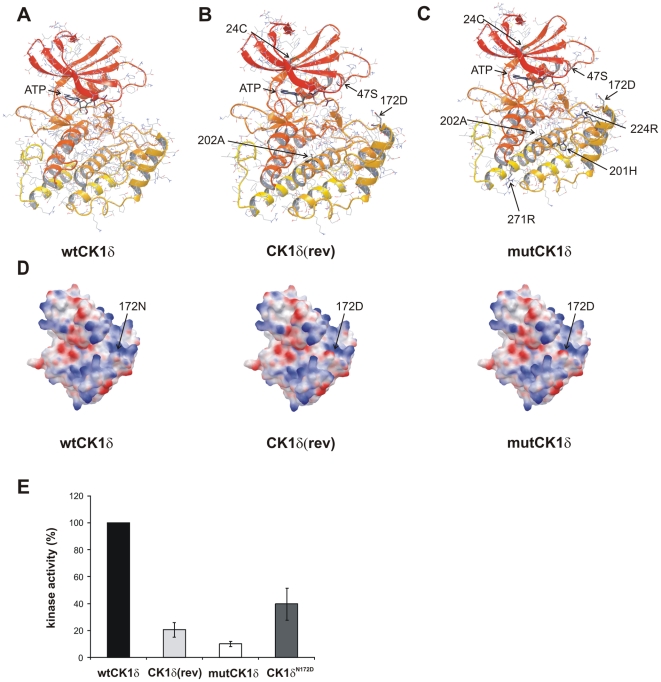
Homology models of rat CK1δ wild-type and mutants. **(A) Homology model (HM) of rat CK1δ containing Mg-ATP in ATP binding pocket (CK1δHM).** **(B) Homology model of rat CK1δ(rev) containing Mg-ATP in ATP binding pocket (CK1δ(rev)).** Mutations compared to wtCK1δ: 24 (Tyr→Cys), 47 (Pro→Ser), 172 (Asn→Asp), 202 (Val→Ala). **(C) Homology model of rat mutCK1δ containing Mg-ATP in ATP binding pocket.** Mutations compared to wtCK1δ: 24 (Tyr→Cys), 47 (Pro→Ser), 172 (Asn→Asp), 201 (Tyr→His), 202 (Val→Ala) 224 (Lys→Arg), 271 (Gln→Arg). **(D) Representation of molecular surface as electrostatic potential for CK1δ homology models.** Color code: neutral (grey), basic (blue) and acidic (red). Significant impact of acidic 172D compared to neutral amide 172N in substrate binding region is highlighted. **(E) Phosphorylation of GST-p53^1–64^ by GST-wtCK1δ, GST-CK1δ(rev), GST-mutCK1δ or GST-CK1δ^N172D^.** *In vitro* kinase assays were performed using GST-p53^1–64^ (FP267) as substrate and GST-wtCK1δ, GST-CK1δ(rev), GST-mutCK1δ or GST-CK1δ^N172D^ as enzyme. The phosphorylated proteins were separated by SDS-PAGE (12.5%) and visualized by Coomassie staining. The degree of phosphorylation was documented by autoradiography as well as Cherenkov counting.

As the structure of the rat CK1δ kinase domain (aa 1–293) has been reported [59], we generated a homology model of mouse CK1δ containing Mg-ATP in the ATP binding pocket (wtCK1δ, figure 2A), representing an active conformation of the kinase domain (the adequate liganded X-ray structure from CK1δ of rat origin is not available). As structural data are not available for the C-terminal domain behind amino acid position 293, our homology models derived from wtCK1δ could only cover mutations from the N-terminus up to amino acid position 293. We introduced the respective mutations into this model (see Materials and Methods) to generate homology models of CK1δ(rev), mutCK1δ, and subsequently minimized the systems using OPLS-2005 force field.

In both, the models of CK1δ(rev) and of mutCK1δ, all mutations were found not to significantly influence the ATP-binding pocket (figure 2B and 2C). Furthermore, compared to the CK1δ original structure (1CSN), the Mg-ATP binding mode was not altered in our models, indicating that the impaired kinase activity of the CK1δ mutants analyzed in this study is not due to impaired ATP binding. As illustrated in figure 2B, CK1δ(rev) mutations 24 (Tyr→Cys), 47 (Pro→Ser) and 202 (Val→Ala) are buried within the protein structure, thereby causing only minor structural differences between wtCK1δ and CK1δ(rev). A similar situation can be found for mutCK1δ regarding mutations 24 (Tyr→Cys), 47 (Pro→Ser), 201 (Tyr→His), 202 (Val→Ala), 224 (Lys→Arg) and 271 (Gln→Arg) (see figure 2C). In contrast, mutation 172 (Asn→Asp) is exposed at the surface of both CK1δ(rev) and mutCK1δ, thereby significantly altering the electrostatic potential of the protein surface from neutral (asparagine/amide) to acidic (aspartic acid) in the substrate binding area of the kinase (figure 2D). Therefore, it is likely that the 172 (Asn→Asp) mutation is responsible for the impaired kinase activity by affecting substrate binding. To test this hypothesis, we exchanged Asn to Asp at position 172 of GST-wtCK1δ to generate GST-CK1δ^N172D^. Analysis of its kinase activity revealed a 40% reduction compared to GST-wtCK1δ, whereas the activity of GST-CK1δ(rev) and GST-mutCK1δ were further reduced to 20 and 10%, respectively (figure 2E).

### Over-expression of CK1δ(rev) and of mutCK1δ in SV-52 cells reduces CK1δ activity and induces reversion of the transformed phenotype by dominant-negative interference with wtCK1δ

**Figure 3:**
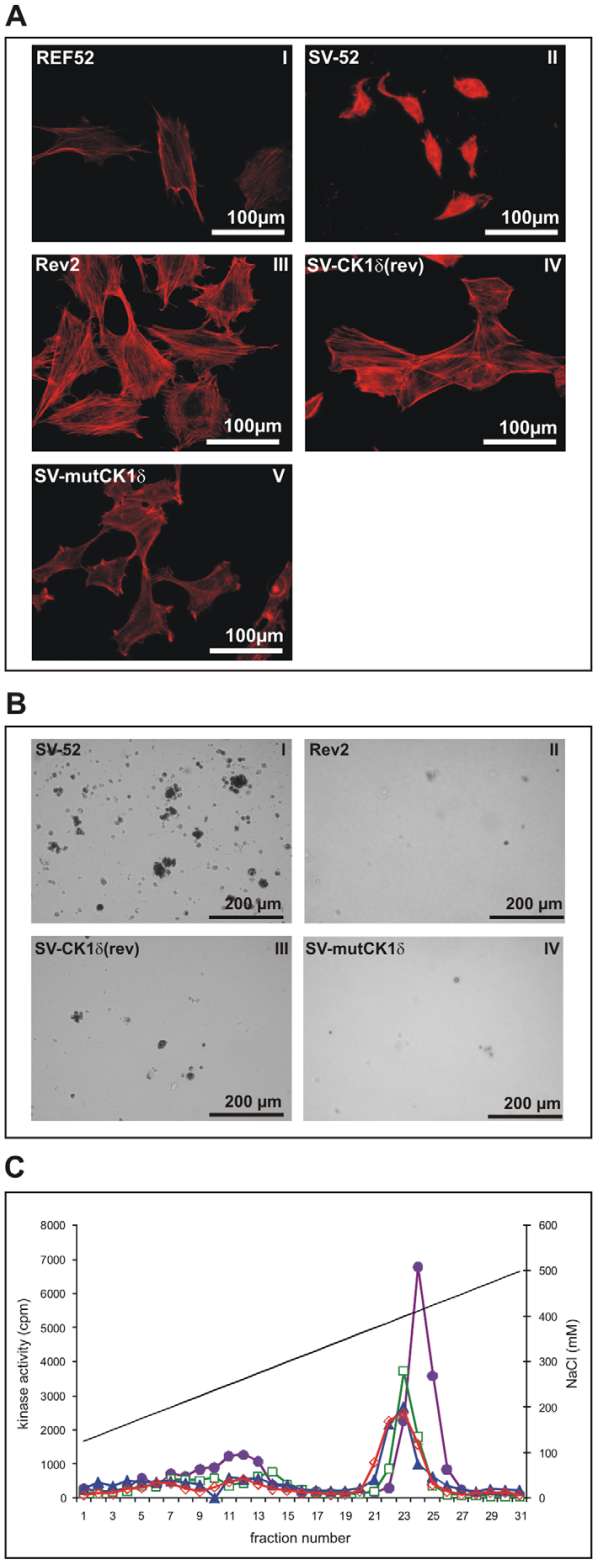
Effects of ectopic expression of CK1δ(rev) or mutCK1δ in SV-52 cells. **(A) Actin network of REF52, SV-52, Rev2, SV-CK1δ(rev) and SV-mutCKδ cells.** The actin cable network of parental REF-52 cells (I), maximal transformed SV-52 cells (II), minimal transformed Rev2 cells (III), SV-CK1δ(rev) and SV-mutCKδ cells was stained with phalloidin-TRITC. **(B) Colony formation of SV-52, Rev2, SV-CK1δ(rev) and SV-CKmutCK1δ cells in soft agar.** Cells were plated in duplicate in culture. Colonies were scored and photographed 20 days after plating (see also table 2). **(C) Detection of CK1 activity in cellular protein fractions derived from anion exchange chromatography.** Soluble extracts of SV-52 (purple, closed circles), Rev2 (blue, closed triangles), SV-CK1δ(rev) (green, open rectangles) and SV-mutCKδ (red, open rectangles) cells were prepared and in each case equal protein amounts were loaded onto a 1 ml Resource Q column. Then proteins were eluted with a linear gradient of increasing NaCl concentration, 0.25 ml fractions were collected, and kinase activity was determined as described in Materials and Methods. The kinase activities in the peak fractions of SV-52, Rev2, SV-CK1δ(rev) and SV-mutCKδ cells were compared.

If the revertant phenotype in Rev2 cells would be causally related to a reduced kinase activity, then ectopic expression of both CK1δ(rev) and mutCK1δ should lead to a reversion of the transformed phenotype in parental SV-52 cells. Indeed, over-expression of either CK1δ(rev) or mutCK1δ in SV-52 cells (SV-CK1δ(rev) and SV-mutCK1δ cells, respectively) resulted in a reversion of the maximal transformed phenotype of SV-52 cells (figure 3A, II) to a minimal transformed phenotype similar to that of Rev2 cells (figure 3A, III), as indicated by a tight actin cable network and a flattened cell shape (figure 3A, IV and V) nearly as distinct as in parental REF52 cells (figure 3A, I). Furthermore, SV-CK1δ(rev) and SV-mutCK1δ cells present reduced cloning efficiency in soft agar (figure 3B, III and IV, and table 2).

**Table 2:** Colony formation and cloning efficiency in soft agar of SV-52, Rev2 and SV-mutCK1δ cells.

cell line	cloning efficiency in soft agar (%)	colony size in soft agar
SV-52	100	large
Rev2	6	microcolony
SV-CK1δ(rev)	9.4	microcolony
SV-mutCK1δ	7	microcolony

Cells were plated in culture dishes as described in Materials and Methods. Colonies were scored and photographed 20 days after plating. The terms “microcolony” and “large” indicate the dominating size type of all established colonies, which only showed limited size variations.

The reversion of the transformed phenotype of SV-52 cells by ectopic expression of CK1δ(rev) and mutCK1δ suggested that these mutant proteins might act in a dominant-negative manner over wtCK1δ. To test this possibility, we first analyzed CK1δ activity in fractionated extracts of SV-CK1δ(rev), SV-mutCK1δ, SV-52 and Rev2 cells. The corresponding kinase activity eluted at 421 mM NaCl (Rev2, SV-CK1δ(rev), and SV-mutCK1δ cells) and 434 mM NaCl (SV-52 cells), respectively (figure 3C). CK1δ activity in SV-CK1δ(rev) cells was reduced to a similar extent as in Rev2 cells compared to SV-52 cells (figure 3C), supporting the conclusions that (i) CK1δ(rev) and mutCK1δ act in a dominant-negative manner over wtCK1δ, and that (ii) a reduced CK1δ activity is important for reversion of the cellular phenotype.

**Figure 4:**
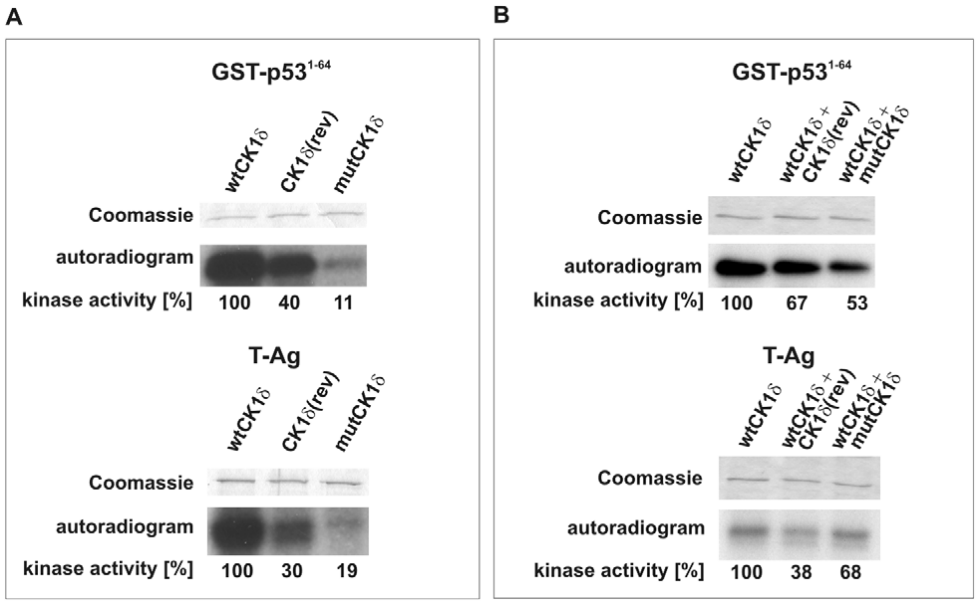
Phosphorylation of GST-p53^1–64^ and baculovirus-expressed T-Ag by GST-CK1δ(rev) and GST-mutCK1δ. **(A) Phosphorylation of GST-p53^1–64^ and baculovirus-expressed T-Ag by GST-wtCK1δ, GST-CK1δ(rev) or GST-mutCK1δ.** *In vitro* kinase assays were performed using GST-p53^1–64^ (FP267) or baculovirus-expressed T-Ag as substrates and GST-wtCK1δ, GST-CK1δ(rev) or GST-mutCK1δ as enzyme. The phosphorylated proteins were separated by SDS-PAGE (12.5%) and visualized by Coomassie staining. The degree of phosphorylation was documented by autoradiography as well as Cherenkov counting. **(B) Phosphorylation of GST-p53^1–64^ or baculovirus-expressed T-Ag by mixed GST-wtCK1δ and GST-CK1δ(rev) or GST-mutCK1δ.** *In vitro* kinases assays were performed using GST-p53^1–64^ (FP267) or baculovirus-expressed T-Ag as substrates and GST-wtCK1δ in combination with equal amounts of either GST-CK1δ(rev) or GST-mutCK1δ as enzyme. In a control reaction, same amounts of GST-wtCK1δ were used diluted in kinase buffer. The phosphorylated proteins were separated by SDS-PAGE (12.5%) and visualized by Coomassie staining. The degree of phosphorylation was documented by autoradiography and by Cherenkov counting and is presented in % activity.

Furthermore, *in vitro* kinase assays revealed a reduction of the catalytic activity of GST-mutCK1δ compared to that of GST-wtCK1δ, as indicated by reduced phosphate incorporation into both, GST-p53^1–64^ and T-Ag (figure 4A). To verify the dominant-negative action of CK1δ(rev) and mutCK1δ by their ability to interact with wtCK1δ, we performed *in vitro* kinase assays using GST-wtCK1δ in combination with equal amounts of GST-CK1δ(rev) or GST-mutCK1δ as enzymes and GST-p53^1–64^ or T-Ag as substrates. As a control reaction, the same amount of GST-wtCK1δ protein (as used above in the mixed kinase reaction) was used in combination with equal amounts of kinase buffer. The data presented in figure 4B demonstrate that addition of either GST-CK1δ(rev) or GST-mutCK1δ to GST-wtCK1δ significantly reduced its ability to phosphorylate GST-p53^1–64^ and T-Ag.

The dominant-negative feature of the cellular alteration in Rev2 revertant cells could be further documented by cell fusion experiments. We fused Rev2 cells firstly with SV-52 cells, and secondly with Rdl1066 cells expressing a C-terminally truncated T-Ag that still exhibits a high transforming activity (75% compared to 100% of wild-type T-Ag [60]). To prove cell fusion, the established fusion cells were characterized regarding SV40 DNA integration sites by Southern blot analyses. Both fusion cell lines (F-SV cells and F-dl1066 cells) contain the parental genomes as indicated by the size of genomic restriction fragments which hybridized to a ^32^P-labeled T-Ag probe (supplementary figure S1A). Furthermore, the expression of the T-Ag proteins from both parental cell lines was confirmed by immunoprecipitation analysis in four individual fusion cell clones (supplementary figure S1B).

F-SV cells and F-dl1066 cells had a well-developed actin cable network similar to those of the parental Rev2H2, Rev Neo and of immortalized REF52 cells (supplementary figure S1C). In contrast, the actin cable networks of parental SV-52zip (supplementary figure S1C) and SV-52 cells (figure 3A) were only weakly developed.

Furthermore, in the fused cells the subcellular localization and the biochemical properties of T-Ag were closely similar to those of T-Ag in Rev2 revertant cells. T-Ag expressed in parental cells is able to interact with the cellular chromatin, whereas this ability is strongly reduced in fusion cells (supplementary figure S1D).

In conclusion, our analyses so far demonstrate that mutant CK1δ variants with reduced kinase activity can revert the maximal transformed phenotype of SV-52 cells by dominant-negative inhibition of wtCK1δ.

### Generation and characterization of mutant CK1δ transgenic mice

To analyze the effects of mutant CK1δ on SV40-induced mammary carcinogenesis *in vivo*, we generated WAP-CK1δ(rev) mice, as described in Materials and Methods, which then could be crossed with WAP-T mice. Analysis of the offspring of WAP-CK1δ(rev) mice for genomic integration and expression of the CK1δ(rev) transgene led to the identification of one transgenic strain (strain G), which showed the highest transgene expression specifically in lactating mammary glands (supplementary figure S2A and B). Sequence analyses of CK1δ(rev) isolated from CK1δ(rev) transgenic animals at backcrosses 10 and 11 revealed three additional mutations in CK1δ(rev) (see second chapter of the result part). This CK1δ mutant then was named mutCK1δ and the respective transgenic animals were called WAP-mutCK1δ transgenic mice.

### Generation and phenotypic characterization of WAP-mutCK1δ/WAP-T mice

To investigate the influence of mutCK1δ on SV40-induced tumorigenesis, animals from strain G (backcross 11) were mated with WAP-T mice (line NP8), which develop mammary carcinomas 5 months after induction (±1.5) with a rate of 83% [20].

#### Survival of mono-transgenic and bi-transgenic mice

**Figure 5:**
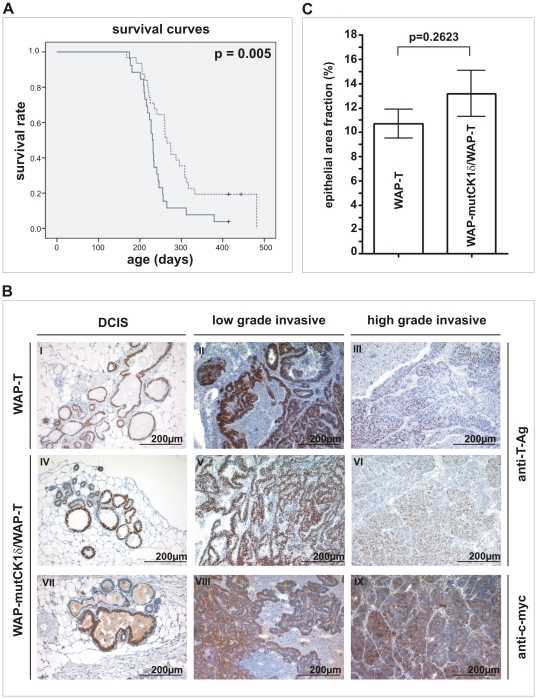
Phenotypic characterization of WAP-mutCK1δ/WAP-T mice. **(A) Survival of induced WAP-T and WAP-mutCK1δ/WAP-T mice.** Kaplan-Meier survival curves show a significant longer life-span of WAP-mutCK1δ/WAP-T mice (median: 265 days) compared to WAP-T mice (median: 230 days). p = 0.005; (monoparous females). <$>\raster="rg1"<$> WAP-T mice; <$>\raster="rg2"<$> WAP-T mice censored, --- WAP-mutCK1δ/WAP-T mice; <$>\raster="rg3"<$> WAP-mutCK1δ/WAP-T mice censored. **(B) T-Ag and mutCK1δ immunostaining of mammary carcinoma in WAP-T transgenic and WAP-mutCK1δ/WAP-T bi-transgenic mice.** Cross-sections of neoplastic mammary glands were immunostained with a polyclonal rabbit antibody against T-Ag (I–VI) and a polyclonal goat antibody against the c-myc epitope tag (VII–IX). Strong nuclear T-Ag staining was detected in DCIS and low grade tumors of WAP-T and WAP-mutCK1δ/WAP-T mice (I, II, IV and V). In high grade tumors, only weak T-Ag expression was found (III, VI). Expression of mutCK1δ, detected by c-myc immunostaining could be found in the cytoplasm and perinuclear region of DCIS (VII) and invasive carcinomas (VIII, IX) of WAP-mutCK1δ/WAP-T mice. **(C) Semi-quantitative evaluation of tumor grading in WAP-mutCK1δ/WAP-T mice.** Epithelial tissue areas of non-invasive carcinomas in mammary glands from day 60 after induction from both transgenic lines were counted as described in Materials and Methods. No significant difference in the number of non-invasive carcinomas in fractions of epithelial areas could be detected.

First, the development of mammary tumors was assessed in parity-induced 28 WAP-mutCK1δ, 26 WAP-T and 31 WAP-mutCK1δ/WAP-T mice. Endpoint analyses revealed that none of the 28 WAP-mutCK1δ females in our study developed a tumor within 16 months of age. We next asked, whether mutCK1δ expression would influence SV40 mammary carcinogenesis in WAP-T mice. Figure 5A shows that bi-transgenic mice had a significantly longer life-span compared to WAP-T mono-transgenic mice (260 d vs. 235 d survival after lactation, respectively; p = 0.005). In addition, 5 out of 31 WAP-mutCK1δ/WAP-T mice (16%) did not develop any tumor, whereas only one out of 26 WAP-T mice (3.6%) remained tumor-free until the age of 16 months.

#### Transgene expression and CK1δ activity in mammary glands and tumors

To verify transgene expression in mammary tumors of WAP-T and WAP-mutCK1δ/WAP-T mice, paraffin sections were immunostained either with a myc-tag specific antibody selectively detecting mutCK1δ, but not wtCK1δ, or a T-Ag specific antibody, respectively. Both, WAP-T and WAP-mutCK1δ/WAP-T mice showed nuclear T-Ag staining in normal gland epithelium. Nuclear T-Ag expression was strong in ductal carcinoma *in situ* (DCIS) and low grade invasive carcinoma (figure 5B I, II, IV, and V), but tended to be reduced in invasive high grade tumors (figure 5B III and VI).

Bi-transgenic mice showed cytoplasmic and perinuclear mutCK1δ immunostaining in normal mammary glands and in all tumors irrespective of tumor grade (figure 5B VII–IX). Co-expression of SV40 T-Ag and mutCK1δ in WAP-mutCK1δ/WAP-T mice renders it likely, that their phenotype as described above is due to the reduced CK1δ activity in these mice.

**Figure 6:**
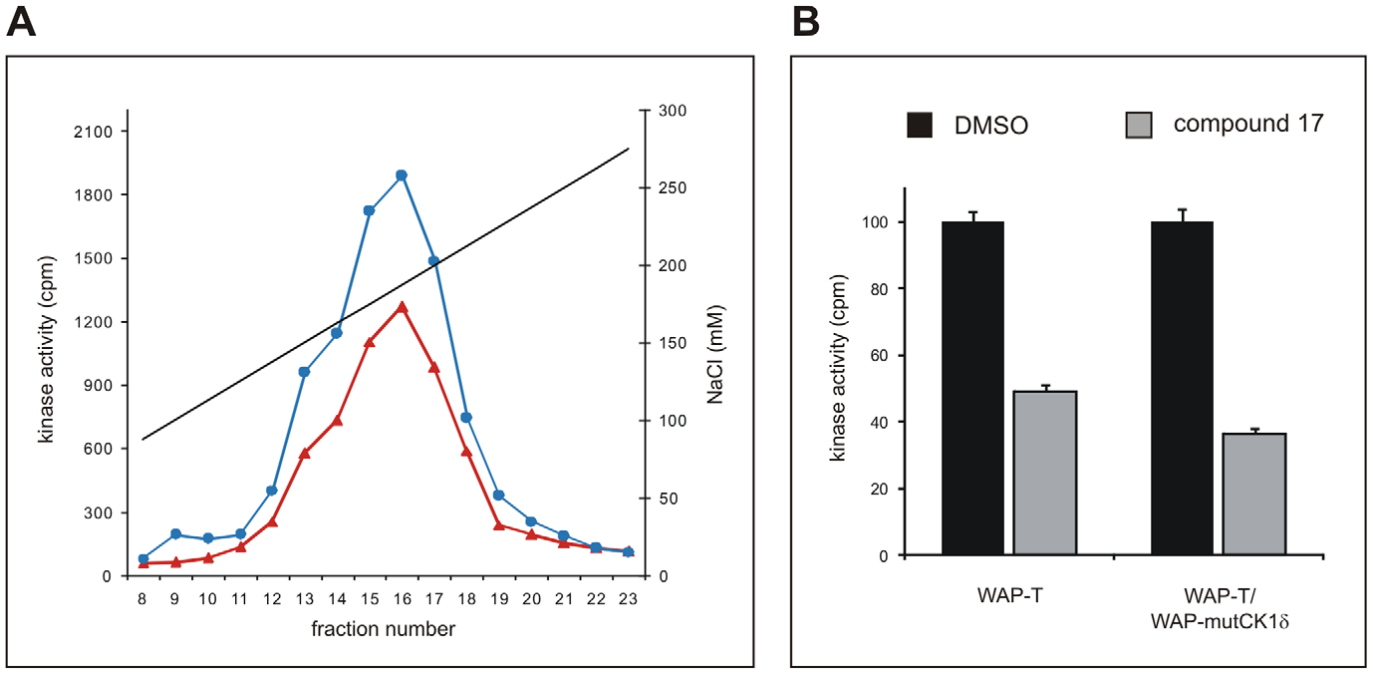
Characterization of CK1 kinase activity in invasive mammary carcinomas of WAP-T and WAP-mutCK1δ/WAP-T mice. **(A) Detection of CK1 activity in mammary carcinoma protein fractions derived from anion exchange chromatography.** Soluble extracts of invasive mammary carcinomas taken from WAP-T transgenic (blue, closed circles) or WAP-mutCK1δ/WAP-T bi-transgenic mice (orange, closed triangles) were prepared and in each case equal protein amounts were loaded onto a 1 ml Resource Q column. Then proteins were eluted with a linear gradient of ascending NaCl concentration. 0.25 ml fractions were collected, and kinase activity was determined as described in Materials and Methods. **(B) Inhibition of CK1 kinase activity in extracts from WAP-T and WAP-mutCK1δ/WAP-T mammary carcinoma tissue using the CK1δ specific inhibitor compound 17.** *In vitro* kinase assays were performed in the presence of 50 nM of inhibitor compound 17 [44] using cellular fractions from fractionated mammary carcinoma protein lysates as source of kinase. Phosphate incorporation into GST-p53^1–64^ was normalized towards DMSO control reactions.

In order to compare CK1δ activity in tumors of WAP-T and WAP-mutCK1δ/WAP-T mice, soluble extracts of invasive mammary tumors of transgenic and bi-transgenic mice were fractionated by anion exchange chromatography as described in Materials and Methods. The kinase activity in the kinase peak fractions of fractionated tumor extracts, eluting between 130 mM and 210 mM NaCl, was reduced by one third in mammary tumor tissue of WAP-mutCK1δ/WAP-T mice compared to that in tumor tissue of WAP-T transgenic mice, when the GST-p53^1–64^ fusion protein was used as substrate (figure 6A). The inhibition of the kinase activity present in the kinase peak fractions by the CK1δ specific small molecule compound 17 [44] (figure 6B) confirmed that CK1δ is the main kinase present in the kinase peak fractions.

#### Clinical staging and histological grading

Both, the clinical staging as well as the histological grading revealed no significant differences between WAP-T mono-transgenic and WAP-mutCK1δ/WAP-T bi-transgenic mice (supplementary figure S3).

#### Semi-quantitative evaluation of tumor initiation frequency in WAP-T and WAP-mutCK1δ/WAP-T mice

The lower frequency and enhanced latency of tumor development in WAP-mutCK1δ/WAP-T mice compared to WAP-T mice could either be due to perturbed tumor initiation, resulting in a lower frequency of hyperplastic lesions and *in situ* carcinomas, or alternatively, by a reduced ability of the tumor cells in DCIS to cross the basal membrane and invade the surrounding tissue. To discriminate between these alternatives, we analyzed histological specimens from mammary glands from both transgenic mouse lines obtained at day 60 after involution, i.e. at a time when hyperplasia and *in situ* carcinoma formation could be observed, but invasive carcinomas had not yet formed (manuscript in preparation). As in the normal involuted mammary gland, fat tissue is most prominent, the epithelial tissue areas in mammary gland tissue of the respective mice is a suitable means to quantitate the formation of such non-invasive carcinomas (see Materials and Methods). The data presented in figure 5C demonstrate that the fraction of epithelial areas is similar in both transgenic mouse lines, thereby indicating similar MIN (mulitfocal interepithelial neoplasia) development in WAP-T and WAP-mutCK1δ/WAP-T mice. Thus tumor initiation is not disturbed in WAP-mutCK1δ/WAP-T mice compared to WAP-T mice. Rather, we assume that in WAP-mutCK1δ/WAP-T mice outgrowth of invasive carcinomas from MIN is affected.

### Reduced CK1δ activity in tumors of WAP-mutCK1δ/WAP-T mice compared to those of WAP-T mice results in an altered expression of genes associated with tumor progression

Outgrowth of invasive carcinomas from MIN in WAP-T mice is a rare event that occurs in a stochastic manner ([61], and manuscript in preparation), and thus is the decisive progression step in malignant mammary carcinogenesis. Therefore, we focused our molecular analysis of tumors from WAP-T and WAP-mutCK1δ/WAP-T mice on two gene sets contained within the RT^2^ “mouse *wnt*-signaling pathway” and “mouse DNA-repair pathways” profiler PCR arrays (see Materials and Methods). Both, *wnt*-signaling and DNA repair are known to be involved in tumor progression and are targets of SV40 early proteins [62] as well as of CK1δ [31]–[38].

Real-Time PCR analysis revealed the up-regulation of 20 genes within the *wnt*- pathway in tumors from WAP-T mice compared to controls (non-tumor tissue), while only six genes were up-regulated in tumors from WAP-mutCK1δ/WAP-T bi-transgenic mice. Six genes from WAP-T tumors and nine genes from WAP-mutCK1δ/WAP-T tumors, respectively, were down-regulated compared to controls. While expression of only three genes within the *wnt*-signaling pathway remained almost unchanged in tumors from WAP-T mice, twelve genes in tumors from WAP-mutCK1δ/WAP-T bi-transgenic mice were similarly expressed compared to controls (table 3). The difference in *gsk3β* expression between the mono- and bi-transgenic tumor samples is striking: *gsk3β* is strongly up-regulated in tumors of mono-transgenic WAP-T mice (more than 30-fold) whereas in tumors of bi-transgenic mice *gsk3β* levels were similar to those of non-tumor samples.

**Table 3:** Analysis of changes in the expression of genes involved in *wnt*-signaling.

		fold change in expression in tumors from	
symbol	description	WAP-T	WAP-mutCK1δ/WAP-T
*aes*	Amino-terminal enhancer of split	3.2 ↑±0.7	1.4 ↑±0.3
*bcl9*	B-cell CLL/lymphoma 9	2.1 ↑±0.5	1.1 ↓±0.7
*btrc*	Beta-transducin repeat containing protein	2.7 ↑±0.07	2.4 ↓±0.04
*ccnd1*	Cyclin D1	5.1 ↓±0.01	2.9 ↓±0.09
*ccnd2*	Cyclin D2	4.0 ↓±0.06	6.3 ↓±0.03
*csnk1a1*	Casein Kinase 1, alpha 1	4.3 ↑±1.2	1.5 ↑±0.04
*csnk2a1*	Casein Kinase 1, alpha polypeptide	3.1 ↓±0.2	1.0 ↑±0.05
*ctbp1*	C-terminal binding protein 1	3.0 ↑±0.5	1.4 ↑±0.3
*ctbp2*	C-terminal binding protein 2	1.6 ↓±0.3	2.0 ↑±0.2
*ctnnb1*	Catenin, beta 1	2.7 ↑±0.3	2.8 ↑±0.9
*dvl2*	Dishevelled, dsh homologe 1 (*Drosophila*)	2.2 ↑±0.4	1.4 ↑±1.2
*fbxw11*	RIKEN cDNA C530030P08 gene	2.9 ↑±0.5	1.4 ↑±0.2
*fbxw4*	F-box und WD-40 domain protein 4	7.7 ↑±1.8	1.9 ↓±0.2
*fzd3*	RIKEN cDNA D130009B15 gene	1.5 ↑±0.2	2.6 ↓±0.2
*gsk3β*	Gylkogen synthase kinase 3 beta	31.6 ↑±11.8	1.2 ↓±0.2
*jun*	Jun oncogene	3.1 ↑±0.5	1.6 ↑±0.3
*nkd1*	Naked cuticle 1 homolog (*Drosophila*)	49.5 ↓±0.02	7.7 ↓±0.03
*nlk*	Nemo like kinase	1.3 ↑±0.04	1.9 ↓±0.05
*ppp2ca*	protein phosphatase 2a, catalytic subunit, alpha isoform	2.0 ↑±0.1	2.3 ↑±0.1
*ppp2r5d*	protein phosphatase 2, regulatory subunit B, delta isoform	3.0 ↑±0.6	1.4 ↑±0.3
*senp2*	SUMO/sentrin specific protease 2	2.9 ↑±0.8	1.5 ↑±0.4
*sfrp1*	secreted frizzled-related sequence protein 1	2.3 ↑±0.4	2.9 ↑±0.5
*tcf7*	Transcription factor 7, T-cell specific	6.4 ↓±0.1	2.1 ↓±0.06
*tle1*	Transducin-like enhancer of split	2.1 ↑±0.3	1.2 ↑±0.4
*wif1*	Wnt inhibitory factor	20.7 ↓±0.04	3.4 ↓±0.1
*wisp1*	WNT1 inducible signaling pathway protein1	1.9 ↑±0.8	7.5 ↑±2
*wnt7b*	Wingless-related MMTV integration site 7B	5.6 ↑±1.5	7.4 ↑±2.3

Total RNA isolated from tumors of WAP-T transgenic and WAP-mutCK1δ/WAP-T bi-transgenic animals was transcribed into complementary DNA. Gene profiling was done using RT^2^ profiler PCR array “mouse *wnt*-signaling pathway” (84 genes) (Superarray SABioscience, Karlsruhe, Germany). The values represent the mean of the observed changes in gene expression in tumors of WAP-T and WAP-mutCK1δ/WAP-T mice compared to the according non-tumor control tissue. Data are presented as ± standard error of the mean (SEM). Increased expression: ↑; decreased expression: ↓.

Analysis of genes of the DNA repair pathway revealed up-regulation of 20 genes and down-regulation of three genes in tumors of mono- and bi-transgenic mice compared to controls (table 4). The only DNA repair gene which was up-regulated (RQ: 5.1) in tumors from WAP-T mice, but down-regulated (RQ: 0.7) in tumors from bi-transgenic mice was *dmc1* (table 4). As a *rad51* related gene, *dmc1* is found at the sites of double strand breaks (DSB) in concert with *rad51*. Interestingly, expression of *rad51* is induced in tumors of both, mono- and bi-transgenic mice (27- and 15-fold, respectively).

**Table 4:** Analysis of changes in the expression of genes involved in DNA repair signaling pathways.

		fold change in expression in tumors from	
symbol	description	WAP-T	WAP-mutCK1δ/WAP-T
***base excision repair (BER)***			
*apex1*	Apurinic/apyrimidinic endonuclease 1	1.7 ↑±0.2	3.1 ↑±0.3
*neil2*	Nei like 2 (*E. coli*)	4.9 ↓±0.04	3.7 ↓±5.7
*neil*3	Nei like 3 (*E. coli*)	4.1 ↑±0.6	15.9 ↑±5.6
*polb*	Polymerase (DNA directed), beta	2.0 ↑±0.2	2.8 ↑±0.9
*ung*	Uracil DNA glycosylase	5.8 ↑±1.2	6.0 ↑±1.2
***nucleotide excision repair (NER)***			
*brip1*	BRCA1 interacting protein C-terminal helicase 1	3.9 ↑±0.6	9.2 ↑±2
*cdk7*	Cyclin-dependent kinase 7	3.2 ↓±0.05	3.2 ↓±0.06
*lig1*	Ligase I, DNA, ATP dependent	4.9 ↑±0.5	7.1 ↑±0.7
*rpa1*	Replication protein A1	3.4 ↑±1.4	3.1 ↑±0.3
*rpa3*	Replication protein A3	5.2 ↑±1.2	9.8 ↑±1.9
***mismatch repair (MMR)***			
*exo1*	Exonuclease 1	8.0 ↑±0.9	34.7 ↑±4.6
*msh5*	MutS homologue 5 (*E. coli*)	3.1 ↓±0.07	5.7 ↓±0.04
*msh6*	MutS homologue 6 (*E. coli*)	2.9 ↑±0.4	2.0 ↑±0.1
*pms1*	Postmeiotic segregation increased 1 (*S. cerevisiae*)	2.5 ↑±0.7	4.4 ↑±0.5
*xrcc6*	X-ray repair complementing defective repair in Chinese hamster cells 6	2.6 ↑±0.3	3.5 ↑±0.2
***double-strand break (DSB) repair***			
*brca1*	Breast cancer 1	5.7 ↑±0.7	17.4 ↑±2.3
*dmc1*	DMC1 dosage suppressor of mck1 homologue	5.1 ↑±2.5	1.4 ↓±0.2
*fen1*	Flap structure specific Endonuclease 1	4.4 ↑±0.7	6.0 ↑±0.7
*rad21*	RAD21 homologue (*S. pombe*)	3.5 ↑±0.3	3.1 ↑±0.4
*rad51*	RAD51 homologue (*S. cerevisiae*)	14.5 ↑±1.9	26.9 ↑±5.5
*rad51c*	Rad51 homologue c (*S. cerevisiae*)	7.0 ↑±1	9.8 ↑±1.2
*rad54l*	RAD54 like (*S. cerevisiae*)	4.1 ↑±0.3	6.2 ↑±1.1
***other genes***			
*rad18*	RAD18 homologue (*S. cerevisiae*)	4.1 ↑±0.5	5.4 ↑±1

Gene profiling was done using RT^2^ profiler PCR array “mouse DNA REPAIR” (84 genes) (Superarray SABioscience, Karlsruhe, Germany). The values represent the mean of the observed changes in gene expression in tumors of WAP-T and WAP-mutCK1δ/WAP-T mice compared to the according non-tumor control tissue. Data are presented as ± standard error of the mean (SEM). Increased expression: ↑; decreased expression: ↓.

## Discussion

We used the system of maximal transformed SV-52 cells and their flat revertants (Rev2) as a tool to analyze the influence of CK1δ on the transformation competence of SV40. Rev2 cells, like their parental SV-52 cells express a genotypically wild-type T-Ag, which is differently phosphorylated in both cell types [11]. In line with published data demonstrating that CK1α and CK1ε, both members of the CK1 kinase family, are able to phosphorylate transformation-relevant sites of T-Ag, we show here that also CK1δ phosphorylates T-Ag *in vitro* and that the activity of CK1δ in revertant Rev2 cells is 2–3-fold decreased compared to its activity in parental SV-52 cells. The reduced CK1δ activity in Rev2 cells is mainly due to point mutations in the coding sequence of CK1δ, as GST-CK1δ(rev) compared to GST-wtCK1δ has a lower capability to phosphorylate T-Ag *in vitro*.

Interestingly, our cell fusion experiments indicated that the Rev2 revertant phenotype is dominant over the SV40 transformed phenotype, suggesting that mutant CK1δ acts in a dominant-negative manner over wtCK1δ. This assumption was strongly supported by our findings that CK1δ(rev) and mutCK1δ proteins, ectopically over-expressed in SV-52 cells were able to confer a Rev2 revertant phenotype to these cells, and that CK1δ(rev) and mutCK1δ were able to reduce the kinase activity of wtCK1δ in *in vitro* kinase assays. The dominant-negative character of CK1δ(rev) and of mutCK1δ can be explained by their ability to interact with wtCK1δ. This assumption is supported by our finding that mixing GST-CK1δ(rev) or GST-mutCK1δ with GST-wtCK1δ inhibited the *in vitro* kinase activity of GST-wtCK1δ. Further experiments based on molecular modeling analyses revealed that a single point mutation (N172D) affecting substrate binding is mainly responsible for the reduced kinase activity of both mutants, CK1δ(rev) and mutCK1δ in comparison to wtCK1δ.

To analyze the influence of an impaired CK1δ activity on SV40-induced tumor formation *in vivo* we established a transgenic mouse model. In this model, CK1δ(rev) was placed under the control of the WAP-promoter to drive transgene expression in ductal and alveolar epithelium of the lactating mammary gland [63]–[65]. The transgenic mouse line with the highest expression of CK1δ(rev) (line G) was selected and back-crossed onto a BALB/c genotype. Sequence analysis of the CK1δ(rev) transgene expressed in transgenic mice at backcrosses 10 and 11 revealed three additional mutations (201 (Tyr→His), 224 (Lys→Arg) and 271 (Gln→Arg); (mutCK1δ)) which, however only slightly affected the mutant CK1δ phenotype of mutCK1δ.

While WAP-mutCK1δ mice had no detectable phenotype, mutCK1δ expression considerably influenced the outcome of SV40-induced mammary carcinogenesis, as WAP-mutCK1δ/WAP-T bi-transgenic mice had a significantly longer life-span than mono-transgenic WAP-T mice. Importantly, five out of 31 induced bi-transgenic mice did not develop tumors at all, whereas only one out of 26 induced WAP-T transgenic mice remained tumor-free. These results are in line with our *in vitro* observations, where the expression of mutCK1δ reversed the phenotypic progression that had occurred in SV-52 cells expressing wtCK1δ. Combined with the finding that both types of mice develop MIN to a rather similar extent, we conclude that MIN/DCIS in bi-transgenic mice have a lower probability to progress to invasive carcinoma. Progression of MIN to invasive carcinomas in WAP-T mice is an extremely rare event, considering that virtually all terminal end buds of all mammary glands develop MIN, while WAP-T mice on the average develop only 2–4 invasive mammary carcinomas ([61], and manuscript in preparation). The even further reduced probability for developing invasive carcinomas in WAP-mutCK1δ/WAP-T bi-transgenic mice thus indicates that CK1δ activity plays an important role in promoting transition of MIN to an invasive carcinoma. However, our finding that histology shows no difference between WAP-T and WAP-mutCK1δ/WAP-T mice suggests that once tumors in bi-transgenic mice had become invasive, they are phenotypically similar to those in WAP-T mice.

Tumor progression induced by SV40 is significantly promoted by activation of the *wnt*-pathway [66], [67], which is also regulated by CK1 family members [36]. The *wnt*-pathway also plays an important role in the development of breast cancer [68]–[71]. Therefore, we performed real-time PCR arrays for analyzing the expression of genes involved in the *wnt*-pathway and observed a strongly enhanced expression of *bcl9*, *fzd3*, *gsk3β*, *jun* and *wif1* in tumors of WAP-T compared to WAP-mutCK1δ/WAP-T mice. As these genes are involved in promoting proliferation, migration and metastasis of tumor cells [72]–[76], and/or in the self-renewal of tumor stem cells in mammary carcinoma [77], we suggest that over-expression of these genes in tumors of WAP-T mice compared to tumors of WAP-mutCK1δ/WAP-T mice might enhance the probability for WAP-T tumors for acquiring an invasive phenotype.

DNA damage response is a candidate anti-cancer barrier in carcinogenesis, as an increased genetic instability is required for the selection of the appropriate “onco-genome”. Within a developing tumor, a fine balance has to be achieved between genetic instability required for tumor progression, and sufficient repair activity required to retain functionality of vital cellular processes. Thus, DNA damage response is activated in many advanced tumors. In SV40-induced *in vitro* transformation and in *in vivo* tumorigenesis genetic instability is achieved by functional inactivation of p53, leading to endoreplication, followed by aneuploidy [78]. Although it is difficult to assign specific consequences to the altered expression of each individual DNA repair gene differently regulated in WAP-T tumors compared to WAP-mutCK1δ/WAP-T tumors, it is a distinct possibility that DNA repair in general is enhanced in WAP-mutCK1δ/WAP-T tumors compared to WAP-T tumors. As a consequence, such enhanced DNA repair could significantly prolong the time required for selecting an appropriate onco-genome, thereby explaining the longer life-span of WAP-mutCK1δ/WAP-T mice.

Mechanistically, the T-Ag/p53 complex plays a pivotal role in SV40-induced tumors in supporting the development of a tumor-associated gene expression profile by its transcriptional activity [79], as well as by inducing genetic instability which allows for the selection of an appropriate onco-genome [80], [81].

In summary, partial inhibition of CK1δ activity reduces the probability of progression of SV40 transformed cells to a maximal transformed phenotype *in vitro*, and prolonged the survival of WAP-mutCK1δ/WAP-T mice. At the molecular level, this inhibition is accompanied by a reduced *wnt*-signaling and an enhanced DNA repair activity in WAP-mutCK1δ/WAP-T mice compared to WAP-T mice.

Our data thus provide evidence that an impaired CK1δ activity influences SV40-induced tumorigenesis, including the modulation of different signaling pathways. Furthermore, our bi-transgenic mouse model presents a suitable tool to identify progression and regression factors involved in carcinogenesis of the mammary ductal carcinoma *in situ*.

## Supporting Information

Data S1 **Additional Methods.**
(DOC)

Figure S1 **Characterization of fusion cells.** **(A) Southern blot analysis of SV40 viral DNA integrated into the genome of SV-52zip, Rev2H2, Rdl1066zip and fusion cells.** Genomic DNA (30 µg) isolated from parental cell lines (SV-52zip, Rev2H2 and Rdl1066zip cells) and from fusion clones (SV-52zip/Rev2H2 (F-SV) or Rdl1066zip/Rev2H2 (F-dl1066) fusion cells) were analyzed for integrated SV40 DNA by Southern blotting. The positions of size markers are indicated. Lanes a and e: Rev2H2; lanes b and d: SV-52zip, lane c: SV-52zip/Rev2H2 fusion clone (F-SV); lane f: Rdl1066zip; lanes g, h, i, j: Rdl1066zip/Rev2H2 fusion clones 1, 14, 9, 13 (F-dl1066 1, 14, 9, 13); m: ^32^P labeled DNA marker; →: T-Antigen specific DNA sequence **(B) Immunoprecipitation of [^35^S]-methionine-labeled T-Ag from Rdl1066zip/Rev2H2 fusion cells (F-dl1066).** T-Ag was immunoprecipitated from cellular lysates of four different fusion clones (lanes a, b, c and d) which had been metabolically labeled with 50 µCi of L-[^35^S]-methionine and L-[^35^S]-cysteine for 1 h. Immunoprecipitates were separated by SDS-PAGE. The expression of both, full length and truncated T-Ag was visualized by autoradiography. **(C) Actin filament staining of REF52, SV-52zip, Rev2H2, Rev Neo, F-SV and F-dl1066 (Rdl1066zip/Rev2H2) cells.** REF52, SV-52zip, Rev2H2, Rev Neo, F-SV and F-dl1066 (Rdl1066zip/Rev2H2) cells were grown on coverslips for two days, fixed, permeabilized and blocked as described in Materials and Methods. The actin network was visualized using TRITC-phalloidin. **(D) Subcellular localization of T-Ag expressed in parental cell lines and fusion clones.** Cells were metabolically labeled with L-[^35^S]-methionine and L-[^35^S]-cysteine before being subfractionated as described in supplementary data file S1. T-Ag was immunoprecipitated using protein A sepharose (Amersham Bioscience, Freiburg, Germany) and the rabbit monoclonal T-Ag specific antibody 108 [82] from SV-52, SV-52zip, Rev2, Rev2H2, Rdl1066, Rdl1066zip, F-SV and F-dl1066 (Rdl1066zip/Rev2H2) cell lysates. The immunoprecipitated proteins were separated on SDS-PAGE, and T-Ag was visualized by fluorography. (N) Cytoplasmic/nucleoplasmic soluble T-Ag; (C) T-Ag extracted from the chromatin; (NM) T-Ag extracted from the nuclear matrix.
(TIF)

Figure S2 **Characterization of mutant CK1δ transgenic mice.** **(A) Transgene expression in lactating mammary glands of WAP-mutCK1δ transgenic mice.** Reverse transcriptase PCR (RT-PCR) analysis, done with RNA isolated from lactating mammary gland tissue (day 5 of lactation) of WAP-mutCK1δ transgenic mice, revealed a transgene expression of mutCK1δ in all 5 transgenic mouse lines. **(B) mutCK1δ immunostaining of lactating mammary glands in WAP-mutCK1δ transgenic mice.** Cross-sections of mammary glands on day 5 of lactation were immunostained with a polyclonal goat antibody against the c-myc epitope tag to analyze the expression pattern of the mutCK1δ transgene. A highly positive cytoplasmic c-myc staining was detected in mammary glands of mouse line C (II) and G (IV), whereas only a weak expression of the transgene was seen in mammary glands of mouse line A (I) and D (III). Mammary glands of line H (V) do not show any c-myc staining. The lactating mammary gland of a non-transgenic littermate served as a control (VI).
(TIF)

Figure S3 **Clinical staging and histological grading of mammary glands and tumors.** **(A) Representative examples of all grades of mammary glands.** Representative examples of all grades of mammary glands of cross-sections from WAP-T (I, III, V, VII) and WAP-mutCK1δ/WAP-T mice (II, IV, VI, VIII). I and II low grade DCIS, III and IV high grade DCIS, V and VI low grade invasive tumor, VII and VIII high grade invasive tumor. All evaluated mammary glands display neoplastic alterations, comprising low and high grade DCIS as well as low and high grade invasive cancer. **(B) Percentage distribution of staging and grading values.** Local tumor stages and histological grades of at least two mammary glands per mouse from each cohort were determined. The largest tumor amongst multiple tumors per mammary gland was staged and graded, respectively.
(TIF)
