# Supplementary material for: Impaired CK1 Delta Activity Attenuates SV40-Induced Cellular Transformation In Vitro and Mouse Mammary Carcinogenesis In Vivo
Source: PLoS One. 2012 Jan 3;7(1):e29709. doi: 10.1371/journal.pone.0029709 (PMC3250488; doi:10.1371/journal.pone.0029709)
Supplement: Data S1 — Additional Methods. (DOC) [file pone.0029709.s001.doc]

# Supplementary data file S1: Additional Methods

**Cell culture (additional cell lines and clones)**

Rdl1066 cells (REF52 cells transformed by the SV40 mutant virus dl1066; [1]), SV-52zip, Rev Neo and Rdl1066zip cells (neomycin resistant SV-52, Rev2 and Rdl1066 cells), Rev2H2 cells (hygromycin resistant Rev2 cells), SV-52zip/Rev2H2 (F-SV cells) and Rdl1066zip/Rev2H2 (F-dl1066 cells) fusion cells were maintained in Dulbecco´s modified Eagle’s medium (DMEM) containing 10% heat-inactivated fetal calf serum (FCS) (Gibco BRL, Karlsruhe, Germany) in a humidified 5% CO_2_ atmosphere. SV-52zip and Rdl1066zip cells were cultivated in the presence of G418 (of 300 µg/ml; PAA, Cölbe, Germany), Rev2H2 cells in the presence of 150 µg/ml hygromycin (Roche, Mannheim, Germany), and F-SV (SV-52zip/Rev2H2) and F-dl1066 (Rdl1066zip/Rev2H2) fusion cells in medium containing G418 and hygromycin, both as specified above.

**Retroviral vectors**

The retroviral vector pZip-TEX [2] mediates resistance to neomycin, whereas the vector pLG90 [3] mediates resistance to hygromycin. Carol Stocking kindly provided psi2-derived pZIPTEX and pLG90 packaging cell lines. Psi2 is a retroviral packaging cell line described in detail by Mann et al. [4].

**Infection and transfection of cells**

Infection of SV-52 cells and Rdl1066 cells with the retroviral vector pZIPTEX and of Rev2 cells with the retroviral vector pLG90 was performed using the calcium phosphate co-precipitation technique [5]. Transfected cells were selected with 400 µg/ml of G418 (SV-52 cells and Rdl1066 cells) or 200 µg/ml of hygromycin B (Rev2 cells). G418 and hygromycin resistant colonies respectively were picked and expanded into cell lines (SV-52zip, Rdl1066zip and Rev2H2).

To establish cell lines stably over-expressing mutant CK1δ isolated from Rev2 cells (CK1δ(rev)) or from WAP-mutCK1δ transgenic mice, SV-52 cells were transfected with pCR3.1-CK1δ(rev) or pcDNA3.1-mutCK1δ, respectively, using the calcium phosphate co-precipitation technique. After selection with 400 µg/ml G418 resistant colonies were picked and expanded into cell lines.

# Cell Fusions

# SV-52zip cells and Rdl1066zip cells, respectively, were fused with Rev2H2 cells applying the polyethylenglycol (PEG) technique [6]. 10^6^ cells of each cell line were washed in fusion medium (F13 medium without calcium and serum). Cells were resuspended in 1 ml of PEG and pre-incubated at 37°C. After 1 min incubation at 37°C, 1 ml of PEG was added, followed by 5 min incubation at 37°C. 1 ml of fusion medium was added, the mixture was incubated for 1 min, followed by successive addition of 2 ml, 4 ml and 8 ml of fusion medium and incubation for 2 min, 4 min and 8 min at 37°C, respectively. Finally, the fusion reaction was stopped by spinning down the cells. The cell pellet was resuspended in 100 ml DMEM/10% FCS, and the cells were plated at a density of 10^6^ cells per 90 mm-diameter dish. Selection with 400 µg/ml G418 and 200 µg/ml hygromycin was started 2 days after plating.

**Metabolic labeling of cells with [^35^S]-methionine**

Cells were metabolically labeled with 50 µCi L-[^35^S] methionine, L-[^35^S] cysteine (trans-label, Hartmann, Germany) for 1 h as described earlier [7].

**Preparation of genomic DNA**

Confluent monolayers of SV-52, SV-52zip, Rev2, Rev2H2 and SV-52/Rev2 fusion cells were lysed overnight at room temperature in 10 mM Tris-HCl [pH 8.0], 10 mM EDTA, 1% sodium dodecylsulfate, 200 µg/ml proteinase K. The lysates were phenol-extracted, and the genomic DNA was isopropanol-precipitated and resuspended in 200 µl 10 mM Tris-HCl [pH 8.0], 1 mM EDTA per 90 mm-diameter dish.

**Southern Blot analysis**

Genomic DNA of SV-52, SV-52zip, Rev2, Rev2H2, and SV-52/Rev2 fusion cells was digested overnight with *Xba*I/*Sst*I or *Xba*I/*Sac*I at 37°C, size-fractionated on 1% agarose gels and alkali-blotted onto Magnacharge nylon membranes (Micron Separations Inc, Westborough, USA) by capillar blotting. The filters were hybridized overnight at 55°C in 5X Denhardt's solution, 0.5% SDS, 5X SSPE, 20 µg/ml denatured herring sperm DNA with ^32^P-labeled T-Ag cDNA probes. Blots were washed twice for 30 min in 1X SSPE, 0.1% SDS at 55°C before being exposed to X-ray films.

***In situ* cell fractionation**

T-Ag was sequentially extracted from different cellular structures using a procedure described elsewhere in detail [8-10]. Prior to preparation of cytoplasmatic/nucleoplasmic soluble extracts, cells were washed in KM buffer (10 mM morpholinepropanesulfonic acid (MOPS) [pH 6.8], 10 mM sodium chloride, 1.5 mM magnesium chloride, 1 mM EGTA, 5 mM DDT, 10% glycerol), followed by cell lysis for 30 min at 4°C in KM buffer containing 1% NP-40, and 20% immunoglobuline free FCS. Chromatin extracts were prepared by digestion of nuclear structures with 100 µg/ml DNAseI (Serva, Heidelberg, Germany) in KM buffer without EGTA and DTT for 15 min at 37°C. After adjustment of the chromatin extraction buffer to 2 M sodium chloride, 1 mM EGTA and 5 mM DDT, the nuclear structures were extracted for additional 30 min at 4°C. Nuclear matrix structures were solubilized in TK buffer (40 mM Tris-HCl [pH 9.0], 5 mM DDT, 10% glycerol) containing 1% Empigen BB (Albright & Wilson, Wetherill Park, Australia) for 60 min at 4°C. All buffers were supplemented with 4 µg/ml leupeptin and 30 µg/ml approtinin. All extracts were adjusted to 150 mM sodium chloride and pH 9.0. In addition, 1% NP-40 was added to the nuclear matrix extract.

**Construction of the WAP-CK1δ(rev) expression vector**

CK1δ isolated from cDNA of Rev2 cells (CK1δ(rev)) was first cloned into the pCR3.1 vector (Invitrogen, Karlsruhe, Germany) [11] and then sublconed in pGEX-2T (Amersham Bioscience, Freiburg, Germany) via *Bam*HI to be expressed as GST fusion protein. In order to add the c-myc epitope tag on the N-terminal site of CK1δ(rev), a PCR reaction using a 5´primer complementary to the c-myc sequence (5’-GAC ACC GGT ACC GAT ATC ATG GAA CAA AAA CTT ATT TCT GAA GAA GAT CTG GAT ATC GGA TCC ATG GAG CTG AGG-3’) and a 3´-primer complementary to the last base pairs of CK1δ (5´-GGATCCTCAGTAGGTGCGTCGTGGGC-3´) was performed. After cloning this construct into the pCR3.1 vector, it was subcloned into the pWAP-T plasmid [12] (kindly provided by A. Graessmann, University of Berlin, Germany), from which the SV40 early region was removed by *Acc*65I/*Bgl*II digest. As a result, CK1δ(rev) was placed under the control of the murine WAP-promoter. Sequencing of CK1δ(rev) revealed the presence of the described mutations.

**Generation and screening of WAP-CK1δ(rev) transgenic mice**

Transgenic mice were generated essentially as described elsewhere (reviewed in [13] ). In short, linear WAP-CK1δ(rev) DNA, excised with *Pvu*II from WAP-CK1δ(rev) plasmid, was microinjected in fertilized C57BL/6-BALB/c hybrid zygotes. The DNA was injected at a concentration of about 2.5 ng/µl in microinjection buffer (10 mM Tris-HCl [pH 7.6], 0.1 mM EDTA), followed by implantation into pseudo-pregnant foster mice. Potential founder animals were identified by PCR of genomic DNA from tail biopsies using a forward primer specific for the myc-tag sequence: 5´-ggtaccatggaggagcagaagctg-3´, and a reverse primer complementary to CK1δ at base pair 837: 5´-tcagcgatggaacagatttctgaagagc-3´, which generates a 878 bp product. PCR was performed as follows: 4 ng/µl genomic DNA, 0.1 µM of each primer, 0.25 mM dNTP-mix, 0.02 U/µl Taq (GE Healthcare, Munich, Germany) and 1X PCR-buffer. PCR program: 95°C for 5 min, followed by 35 cycles of 30 s at 95°C, 30 s at 62°C and 1 min at 72°C followed by an additional 5 min at 72°C.

Resulting WAP-CK1δ(rev) transgenic mice were continuously backcrossed as hemizygotes on the BALB/c genetic background. At backcrosses 10 and 11 RNA was isolated from transgenic mice and transcribed into cDNA. Sequencing of PCR products revealed the presence of three additional mutations at base pairs 601 (CAC → TAC), 671 (AAG → AGG) and 812 (CAG → CGG) leading to amino acid mutations at positions 201 (Y→H), 224 (K→R) and 271 (Q→R) of CK1δ(rev). From now on WAP-CK1δ(rev) transgenic mice were called WAP-mutCK1δ transgenic mice.

**Reverse transcription PCR (RT-PCR)**

Expression of the mutCK1δ transgene in mammary gland tissue was analyzed using the primer pair used for genotyping of WAP-CK1δ(rev) mice (see above) and cDNA generated from extracted mammary gland RNA (see Materials and Methods).

**Cloning of mutCK1δ**

CK1δ amplified (5´-Primer 5´-GGATCCATGGAGCTGAGGGTCGGGAATAG-3´ and 3´-Primer 5´-GGATCCTCAGTAGGTGCGTCGTGGGC-3´) from genomic DNA of WAP-CK1δ(rev) (later: WAP-mutCK1δ) transgenic mice was first cloned into the pcDNA3.1 vector (Invitrogen, Karlsruhe, Germany) and later subcloned into the pGEX-2T (Amersham Biosience, Freiburg, Germany) vector via *Bam*HI for being expressed as GST fusion protein. In order to add the HA-epitope tag on the N-terminal site of mutCK1δ, a PCR reaction using a 5´-primer containing the sequence of the HA-tag and the first base pairs of CK1δ (5´‑GATATCATGTACCCATACGATGTTCCAGATTACGCTCTTCATATGGCGATGGAGCTGAGG-3´) and a 3´primer complementary to the last base pairs of CK1δ (5´-GGATCCTCAGTAGGTGCGTCGTGGGC-3´) was performed. The PCR product was cloned into the pcDNA3.1 vector (Invitrogen, Karlsruhe, Germany). All plasmids were sequenced before use to confirm the presence of the described mutations.

**Generation of mutCK1δ^N172D^ via site directed mutagenesis**

Wild type CK1δ, expressed in the pGEX-2T vector was used as template for introducing one additional mutation into CK1δ at base pair 514 (AAC → GAC) (amino acid position 172 N→D) by site directed mutagenesis using the Quick Change site directed mutagenesis kit from Agilent Technologies (Böblingen, Germany). Mutagenesis was performed as described in the manufacturer’s protocol using following primers: 5´-Primer: 5´-ATCCCCTATCGAGAGGACAAGAACCTCACAGG-3´, 3´-Primer: 5´-TAGGGGATAGCTCTCCTGTTCTTGGAGTGTCC-3´.

**Clinical tumor staging and histological tumor grading**

To compare tumor development between mono- and bi-transgenic mouse lines, a clinical staging and a histological grading system was used based on the Annapolis consensus conference on mammary pathology of genetically engineered mice [14].

For macroscopic staging, the mammary glands were designated by numbers on the left body side in rostral-caudal direction (1, cervical; 2, thoracal; 3, abdominal; 4, inguinal) and on the right body side in caudal-rostral direction (5, inguinal; 6, abdominal; 7, thoracal; 8, cervical). Stage 0, no macroscopic abnormality detected (NAD); Stage 1, diffusely thickened mammary gland; Stage 2, small solid nodules (up to 0.2 cm); Stage 3, tumor 0.3 – 0.8 cm; Stage 4, tumor < 1.6 cm; Stage 5, tumor > 1.6 cm [15].

Transgenic T-Ag expression by itself causes nuclear enlargement and a marked mitotic rate of the mammary glandular epithelium. It thus influences parameters that are used to grade human DCIS and invasive breast cancer, respectively. For this reason, the standard grading systems of human mammary carcinoma were not applicable in our mouse model. We therefore designed a two-tier system defining high grade by cytological criteria, i.e. nuclear pleomorphism, nuclear hyperchromasia, and by tumor architecture, i.e. the extent of intraductal proliferation (DCIS), the absence of tubular/glandular growth (invasive cancer), and the degree of necrosis.

**References**

1. Pipas JM, Peden KW, Nathans D (1983) Mutational analysis of simian virus 40 T antigen: isolation and characterization of mutants with deletions in the T-antigen gene. Mol Cell Biol 3: 203-213.

2. Brown M, McCormack M, Zinn KG, Farrell MP, Bikel I, et al. (1986) A recombinant murine retrovirus for simian virus 40 large T cDNA transforms mouse fibroblasts to anchorage-independent growth. J Virol 60: 290-293.

3. Gritz L, Davies J (1983) Plasmid-encoded hygromycin B resistance: the sequence of hygromycin B phosphotransferase gene and its expression in Escherichia coli and Saccharomyces cerevisiae. Gene 25: 179-188.

4. Mann R, Mulligan RC, Baltimore D (1983) Construction of a retrovirus packaging mutant and its use to produce helper-free defective retrovirus. Cell 33: 153-159.

5. Graham FL, van der Eb AJ (1973) Transformation of rat cells by DNA of human adenovirus 5. Virology 54: 536-539.

6. Davidson RL, Park G (1976) Improved techniques for the induction of mammalian cell hybridization by polyethylene glycol. Som Cell Genet 2: 165-176.

7. Knippschild U, Kiefer J, Patschinsky T, Deppert W (1991) Phenotype-specific phosphorylation of simian virus 40 tsA mutant large T antigens in tsA N-type and A-type transformants. J Virol 65: 4414-4423.

8. Deppert W, Haug M, Steinmayer T (1987) Modulation of p53 protein expression during cellular transformation with simian virus 40. Mol Cell Biol 7: 4453-4463.

9. Staufenbiel M, Deppert W (1983) Different structural systems of the nucleus are targets for SV40 large T antigen. Cell 33: 173-181.

10. Staufenbiel M, Deppert W (1984) Preparation of nuclear matrices from cultured cells: subfractionation of nuclei in situ. J Cell Biol 98: 1886-1894.

11. Wolff S, Xiao Z, Wittau M, Sussner N, Stoter M, et al. (2005) Interaction of casein kinase 1 delta (CK1 delta) with the light chain LC2 of microtubule associated protein 1A (MAP1A). Biochim Biophys Acta 1745: 196-206.

12. Tzeng YJ, Guhl E, Graessmann M, Graessmann A (1993) Breast cancer formation in transgenic animals induced by the whey acidic protein SV40 T antigen (WAP-SV-T) hybrid gene. Oncogene 8: 1965-1971.

13. Hogan B, Costantini F, Lacy E (1986) Manipulating the mouse embryo: A laboratory manual. New York: Cold Spring Harbor Laboratory. 332 p.

14. Cardiff RD, Anver MR, Gusterson BA, Hennighausen L, Jensen RA, et al. (2000) The mammary pathology of genetically engineered mice: the consensus report and recommendations from the Annapolis meeting. Oncogene 19: 968-988.

15. Heinlein C, Krepulat F, Lohler J, Speidel D, Deppert W, et al. (2008) Mutant p53(R270H) gain of function phenotype in a mouse model for oncogene-induced mammary carcinogenesis. Int J Cancer 122: 1701-1709.
