# Supplementary figures and images for: Impaired CK1 Delta Activity Attenuates SV40-Induced Cellular Transformation In Vitro and Mouse Mammary Carcinogenesis In Vivo
Source: PLoS One. 2012 Jan 3;7(1):e29709. doi: 10.1371/journal.pone.0029709 (PMC3250488; doi:10.1371/journal.pone.0029709)

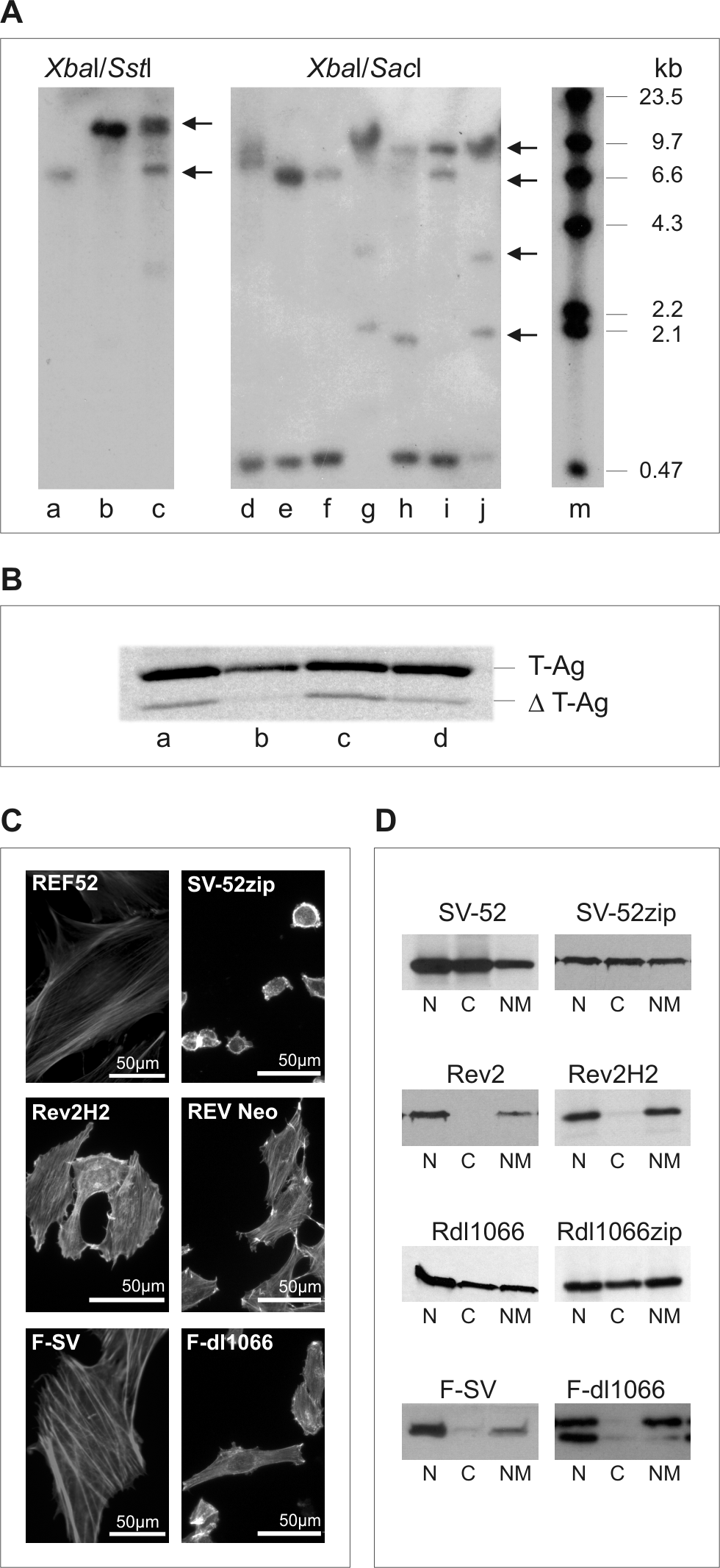

Supplement: Figure S1 — Characterization of fusion cells. (A) Southern blot analysis of SV40 viral DNA integrated into the genome of SV-52zip, Rev2H2, Rdl1066zip and fusion cells. Genomic DNA (30 µg) isolated from parental cell lines (SV-52zip, Rev2H2 and Rdl1066zip cells) and from fusion clones (SV-52zip/Rev2H2 (F-SV) or Rdl1066zip/Rev2H2 (F-dl1066) fusion cells) were analyzed for integrated SV40 DNA by Southern blotting. The positions of size markers are indicated. Lanes a and e: Rev2H2; lanes b and d: SV-52zip, lane c: SV-52zip/Rev2H2 fusion clone (F-SV); lane f: Rdl1066zip; lanes g, h, i, j: Rdl1066zip/Rev2H2 fusion clones 1, 14, 9, 13 (F-dl1066 1, 14, 9, 13); m: 32P labeled DNA marker; →: T-Antigen specific DNA sequence (B) Immunoprecipitation of [35S]-methionine-labeled T-Ag from Rdl1066zip/Rev2H2 fusion cells (F-dl1066). T-Ag was immunoprecipitated from cellular lysates of four different fusion clones (lanes a, b, c and d) which had been metabolically labeled with 50 µCi of L-[35S]-methionine and L-[35S]-cysteine for 1 h. Immunoprecipitates were separated by SDS-PAGE. The expression of both, full length and truncated T-Ag was visualized by autoradiography. (C) Actin filament staining of REF52, SV-52zip, Rev2H2, Rev Neo, F-SV and F-dl1066 (Rdl1066zip/Rev2H2) cells. REF52, SV-52zip, Rev2H2, Rev Neo, F-SV and F-dl1066 (Rdl1066zip/Rev2H2) cells were grown on coverslips for two days, fixed, permeabilized and blocked as described in Materials and Methods. The actin network was visualized using TRITC-phalloidin. (D) Subcellular localization of T-Ag expressed in parental cell lines and fusion clones. Cells were metabolically labeled with L-[35S]-methionine and L-[35S]-cysteine before being subfractionated as described in supplementary data file S1. T-Ag was immunoprecipitated using protein A sepharose (Amersham Bioscience, Freiburg, Germany) and the rabbit monoclonal T-Ag specific antibody 108 [82] from SV-52, SV-52zip, Rev2, Rev2H2, Rdl1066, Rdl1066zip, F-SV and F-dl1066 (Rdl1066zip/Rev2H2 [file pone.0029709.s002.tif]

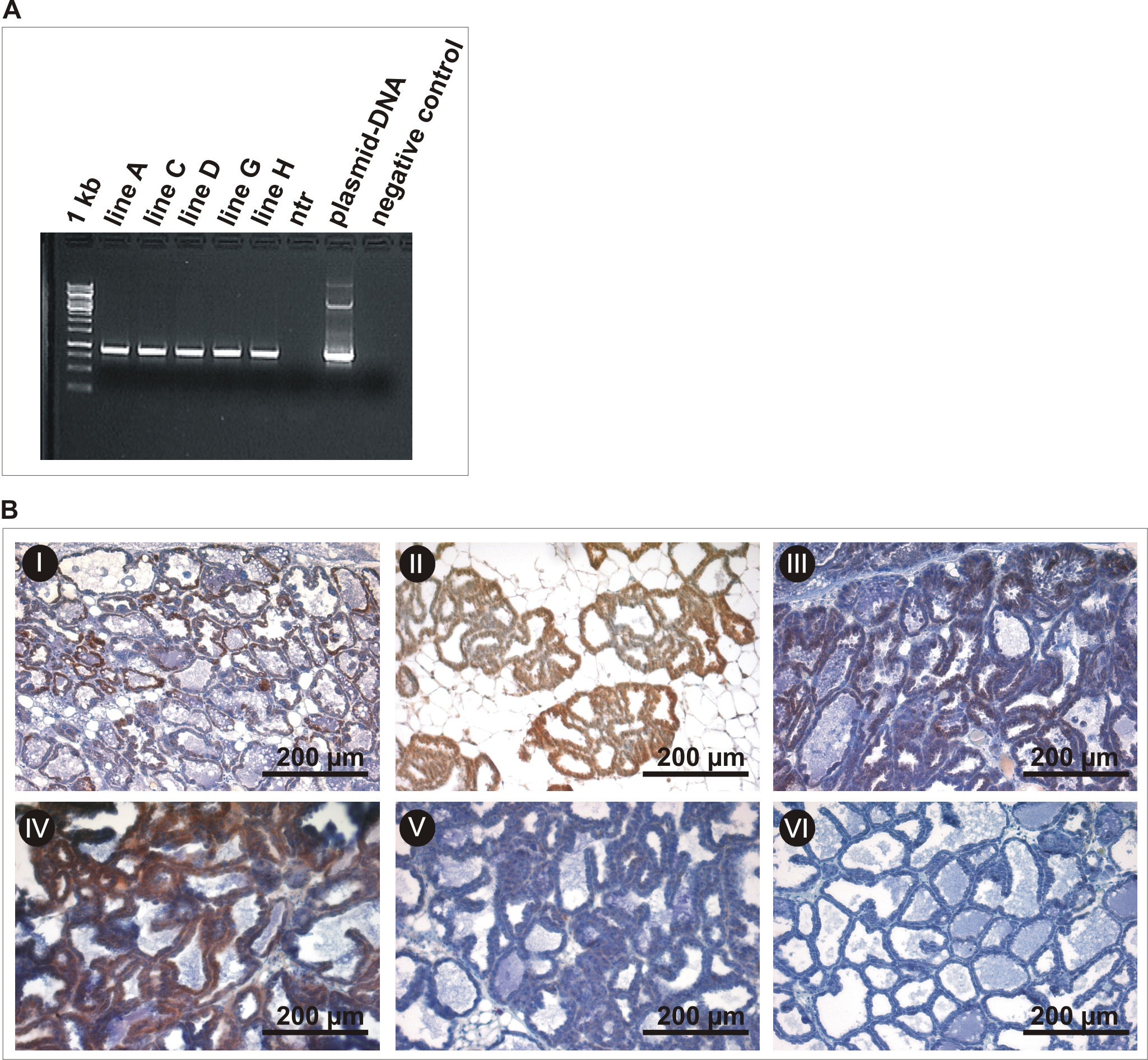

Supplement: Figure S2 — Characterization of mutant CK1δ transgenic mice. (A) Transgene expression in lactating mammary glands of WAP-mutCK1δ transgenic mice. Reverse transcriptase PCR (RT-PCR) analysis, done with RNA isolated from lactating mammary gland tissue (day 5 of lactation) of WAP-mutCK1δ transgenic mice, revealed a transgene expression of mutCK1δ in all 5 transgenic mouse lines. (B) mutCK1δ immunostaining of lactating mammary glands in WAP-mutCK1δ transgenic mice. Cross-sections of mammary glands on day 5 of lactation were immunostained with a polyclonal goat antibody against the c-myc epitope tag to analyze the expression pattern of the mutCK1δ transgene. A highly positive cytoplasmic c-myc staining was detected in mammary glands of mouse line C (II) and G (IV), whereas only a weak expression of the transgene was seen in mammary glands of mouse line A (I) and D (III). Mammary glands of line H (V) do not show any c-myc staining. The lactating mammary gland of a non-transgenic littermate served as a control (VI). (TIF) [file pone.0029709.s003.tif]

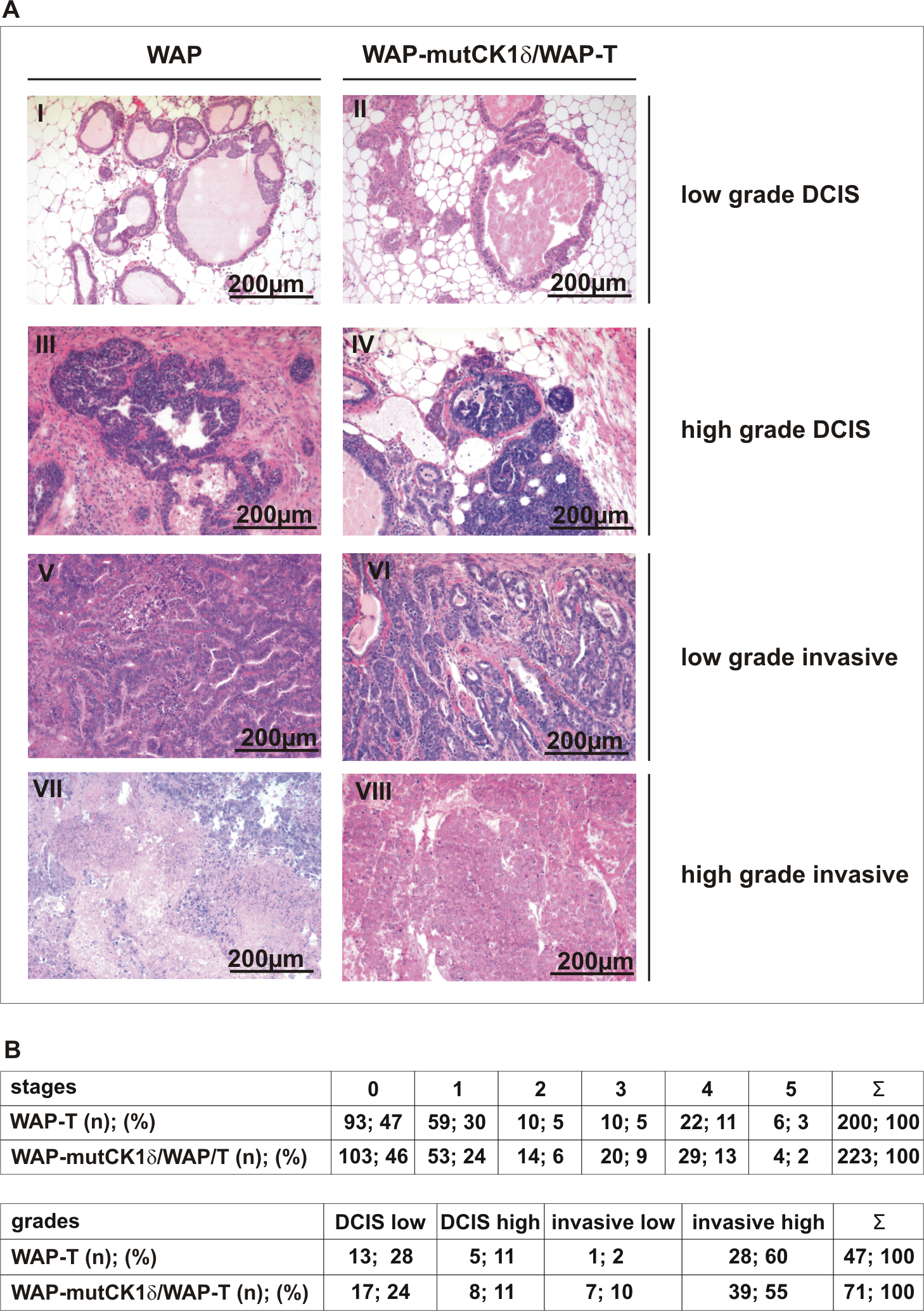

Supplement: Figure S3 — Clinical staging and histological grading of mammary glands and tumors. (A) Representative examples of all grades of mammary glands. Representative examples of all grades of mammary glands of cross-sections from WAP-T (I, III, V, VII) and WAP-mutCK1δ/WAP-T mice (II, IV, VI, VIII). I and II low grade DCIS, III and IV high grade DCIS, V and VI low grade invasive tumor, VII and VIII high grade invasive tumor. All evaluated mammary glands display neoplastic alterations, comprising low and high grade DCIS as well as low and high grade invasive cancer. (B) Percentage distribution of staging and grading values. Local tumor stages and histological grades of at least two mammary glands per mouse from each cohort were determined. The largest tumor amongst multiple tumors per mammary gland was staged and graded, respectively. (TIF) [file pone.0029709.s004.tif]
